# Nanometric and Hydrophobic Green Rust Minerals upon Exposure to Amino Acids and Nickel as Prerequisites for a Primitive Chemiosmosis

**DOI:** 10.3390/life15040671

**Published:** 2025-04-19

**Authors:** Nil Gaudu, Chloé Truong, Orion Farr, Adriana Clouet, Olivier Grauby, Daniel Ferry, Philippe Parent, Carine Laffon, Georges Ona-Nguema, François Guyot, Wolfgang Nitschke, Simon Duval

**Affiliations:** 1 Laboratoire de Bioénergétique et Ingénierie des Protéines (BIP), Aix-Marseille Université, UMR 7281 IMM-CNRS, 31 Chemin Joseph Aiguier, 13402 Marseille, France; ctruong@imm.cnrs.fr (C.T.); orionfarr@gmail.com (O.F.); aclouet@imm.cnrs.fr (A.C.); nitschke@imm.cnrs.fr (W.N.); sduval@imm.cnrs.fr (S.D.); 2 Centre Interdisciplinaire des Nanosciences de Marseille (CINaM), Aix-Marseille Université, UMR 7325 CNRS, Campus de Luminy, 13288 Marseille, France; olivier.grauby@univ-amu.fr (O.G.); daniel.ferry@cnrs.fr (D.F.); philippe.parent@univ-amu.fr (P.P.); carine.laffon@univ-amu.fr (C.L.); 3 Institut de Minéralogie, de Physique des Matériaux et de Cosmochimie (IMPMC), Sorbonne Université, UMR 7590 CNRS, 4 Place Jussieu, 75005 Paris, France; georges.ona_nguema@sorbonne-universite.fr (G.O.-N.);

**Keywords:** alkaline hydrothermal vents, fougerite, emergence of life, chemiosmosis, amino acids, adsorption, hydrophobicity

## Abstract

Geological structures known as alkaline hydrothermal vents (AHVs) likely displayed dynamic energy characteristics analogous to cellular chemiosmosis and contained iron-oxyhydroxide green rusts in the early Earth. Under specific conditions, those minerals could have acted as non-enzymatic catalysts in the development of early bioenergetic chemiosmotic energy systems while being integrated into the membrane of AHV-produced organic vesicles. Here, we show that the simultaneous addition of two probable AHV components, namely nickel and amino acids, impacts green rust’s physico-chemical properties, especially those required for its incorporation in lipid vesicle’s membranes, such as decreasing the mineral size to the nanometer scale and increasing its hydrophobicity. These results suggest that such hydrophobic nano green rusts could fit into lipid vesicle membranes and could have functioned as a primitive, inorganic precursor to modern chemiosmotic metalloenzymes, facilitating both electron and proton transport in early life-like systems.

## 1. Context

Living systems are extremely far from thermodynamic equilibrium and hence represent states of extraordinarily low entropy. As recognized by Erwin Schrödinger more than half a century ago [1], in order to obey the second law of thermodynamics, life’s low entropy state must be maintained by the influx of free energy from the environment, thereby taking the entire system (life plus its environment) closer to thermodynamic equilibrium. Hypotheses pretending to rationalize the origin of life fall into one of two categories. Those belonging to the first category envisage that the foundational ordered state able to extract free energy from the environment arose by chance [2,3] from a mixture of organic molecules (frequently called “building blocks-first hypotheses”) while those of the second one consider that already at life’s inception, ordering and structuring was driven by environmental free energy and that life’s order therefore never came from disorder [1] but actually corresponded to some sort of dissipative formation of low entropy states [4,5,6,7,8,9,10]. These latter hypotheses are, therefore, “free-energy-metabolism-first” scenarios.

Given the (literally) vanishingly small probability of the degree of spontaneous entropy decrease required to fortuitously bring about a metabolizing ensemble of building blocks, we adhere to the second option, as does an increasing number of researchers interested in the emergence of life [8,9,10,11,12,13,14]. This raises the question of which mechanism of environmental free energy may have been converted into the spatial and temporal order inherent to life. While several ad hoc scenarios have been proposed [12,15,16,17,18,19], our strategy is guided by the synthesis of a (biological) top-down approach and (palaeogeochemical) bottom-up considerations as detailed in the following.

### 1.1. Top-Down; From Extant Life Back to the LUCA

The driving environmental type of free energy for all of extant life is redox disequilibria of various sorts (in the case of photosynthetic organisms generated by photon-induced charge-separations) [20]. The main mechanism for converting such redox disequilibria into intracellular chemical-free energy is the bioenergetic process of chemiosmosis [21]. Chemiosmosis is a two-step conversion process, first transducing redox disequilibria into cation-motive potentials across the cytoplasmic membrane. These cation-motive potentials are in a second step converted into the chemical free energy contained in far-from-equilibrium ratios of polyphosphates to phosphates [21,22]. Phylogenetic analyses of a plethora of enzymes involved in chemiosmosis indicate that this mechanism represented life’s bioenergetic principle at least since the times of the last universal common ancestor (LUCA), considered to have existed more than 3.5 billion years ago [23,24]. We therefore consider conceivable that, rather than being a late-evolved process, chemiosmosis driven by redox tensions may have been life’s primordial mode of free energy conversion since the earliest days [25].

### 1.2. Bottom-Up; From Palaeogeochemistry Towards the LUCA

**Figure 1:**
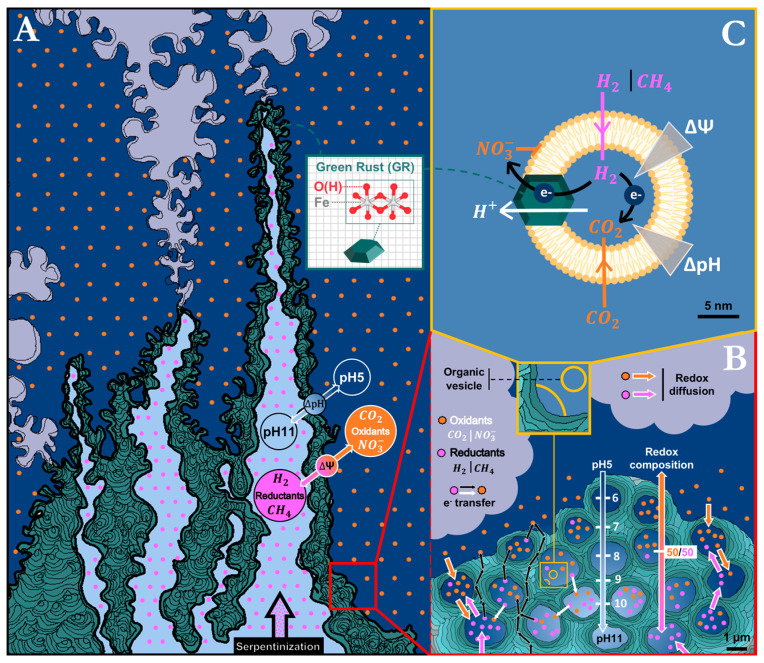
Depiction of the alkaline hydrothermal vent (AHV) setup close to life’s emergence displaying: (**A**) Overall physico-chemical and mineralogical properties of the vent: an internal reducing alkaline fluid (light blue fluid) resulting from serpentinization processes and containing reductants such as H_2_ and CH_4_ (pink dots) at an alkaline pH value of about 11 circulates upwards and mixes with the external weakly acidic (pH 5) and oxidizing ocean (dark blue fluid) containing oxidants such as CO_2_ and NO_3_^−^ (orange dots) as well as high iron concentrations, leading to the precipitation of mainly iron oxy-hydroxides such as green rust. This precipitate forms the chimney walls separating the internal reducing alkaline fluid from the external weakly acidic oxidizing seawater, thus building up apparent ΔpH (dark to light blue) and ∆Ψ (orange to pink) gradients over the width of the wall. (**B**) A closer look at the microporous network within the chimney walls: the apparent large-scale ΔpH and ∆Ψ of (A) cannot prevail over the µm-sized micropore membranes as detailed in the text (Section 1.3) and as briefly summarized as being due to the following:—the progressive mixing of proton concentrations and redox species across micropores as a result of the diffusion of oxidants and reductants (orange and pink arrows) and fluids through the mineral structures.—the high conductivity of the mineral barriers forming the micropores (see main text). (**C**) The scenario of organic vesicles formed as a result of mineral-mediated synthesis of hydrocarbons within the mound: under such conditions, a nanometric, hydrophobic green rust crystal may become embedded in the nanometer-sized vesicle membrane providing the mechanistic seat for proto-chemiosmotic processes based on the redox-driven formation of cross-membrane ΔpH and ∆Ψ to occur.—The free diffusion of gaseous reductants toward the internal volume of the vesicles, together with the exclusion of charged oxidizing solutes such as nitrate and carbonate from the internal compartments, provides cross-barrier redox disequilibria.—At the interface of the two compartments, green rust would ensure the catalysis of redox reactions between internal H_2_/CH_4_ and external NO_3_^−^ through cross-membrane electron transfer coupled to proton movement, thus building pH and electrostatic (∆Ψ) gradients.—Simultaneously, the internal redox reactivity of H_2_/CH_4_ with the freely diffusing CO_2_ may initiate the formation and concentration of organic molecules within the vesicle.

In the early 1980s, submarine alkaline hydrothermal vents on the early Earth were proposed as promising locales for the emergence of life [11]. Characteristic chimneys harbor microporous mineral structures initially observed on fossil chimneys located in a mine in Ireland. Subsequently, the same observations were reproduced in active hydrothermal fields such as Lost City in the mid-Atlantic Ocean [26,27,28] and the Shinkai Seep close to the Mariana Trench [29], wherein labyrinthine networks of interconnected micropores precipitate from the alkaline vents fluid mixing with the weakly acid seawater (Figure 1A) [30]. Initially, the alkaline vent chimneys on the early Earth were considered to contain iron-, nickel- and sulfide-based minerals such as greigite and mackinawite, together with silicates and carbonates [31,32,33]. Laboratory experiments subsequently suggested that, due to limited concentrations of sulfides, these minerals were likely only partial constituents of chimney walls [34]. Analyses of the present-day alkaline hydrothermal Shinkai Seep field in the Pacific Ocean indeed found a type of chimney mainly made up of brucite, a Mg-containing layered double hydroxide (LDH) belonging to the class of anionic clays [30]. Accounting for the fact that modern, oxygenated ocean waters contain very low amounts of dissolved iron while the anoxic Hadean and Archaean oceans were iron-rich [31,35], it follows that primordial chimneys likely contained ferrous and ferric iron ions, yielding iron oxy-hydroxides, the reactivity of which exceeds that of carbonate species, turning them into the main inorganic catalysts at the vent/ocean interface.

More particularly, these conditions yield the LDH-mineral fougerite [36], the naturally occurring form of the synthetic mineral green rust (GR) known to material sciences since the 1950s [37] (Figure 1A).

The effluents from submarine hydrothermal vents developed on serpentinization systems are not only strongly alkaline but also highly reducing due to the presence of H_2_ and CH_4_ [12,19]. In contrast, the weakly acidic primordial ocean contained substantial concentrations of oxidants such as CO_2_ (in equilibrium with its solvatation products HCO_3_^−^ and CO_3_^2−^) and nitrate [19,38,39,40,41]. Both the redox and the pH gradient were likely maintained by the venting fluid at the sites of these chimneys over tens of thousands of years [12,42,43], steadily and consistently providing sources of free energy [19,44]. Intriguingly, the redox compounds present in the alkaline vents (i.e., H_2_, CH_4_, CO_2_, NO_3_^−^) are also common electron donors and acceptors for bioenergetic processes in extant life. The free energy characteristics of alkaline hydrothermal vents were therefore early pointed out as strikingly reminiscent of chemiosmotic systems [12], arguing again, but from an entirely different perspective, for the possibility of chemiosmosis-related processes having represented life’s earliest free energy metabolism (Figure 1A).

### 1.3. The Gap Between Alkaline Hydrothermal Vents and Actual Chemiosmosis

Despite similarities between the free energy landscape of alkaline hydrothermal vents and extant bioenergetics, the presumed transition from the former to the latter is far from straightforward. The mineral walls separating micro-compartments [18,45,46] within the microporous chimneys, proposed to have been precursors to the cytoplasmic membranes, are unsuitable for being the seat of chemiosmotic processes since they differ from chemiosmosis-bearing membranes in three crucial parameters: thickness, relative permittivity (also called dielectric constant), and conductivity (the latter two being related, see below) (Figure 1B).

A chemiosmotic cell can be idealized as a spherical capacitor that gets charged (negative inside and positive outside) by the bioenergetic electron transport system, yielding an electrostatic potential (U) proportional to the charge-surficial density (C = charge/area) and to the thickness of the dielectric membrane (d) but inversely proportional to the permittivity (ε) according to U = Cd/ε. Energized cells typically generate electrostatic potentials in the range of 200 mV between the two faces of the membrane [47]. The electrostatic potential, in turn, yields a force pulling cations (mostly protons but in certain cases also sodium cations) from the outside towards the inner volume. The strength and direction of this force (F) is proportional to the electric field, i.e., the gradient of the electrostatic potential (F~gradU or F~∇U).

Mineral barriers in vent chimneys are generally µm-sized, while the thickness of a chemiosmotic membrane is in the range of 5 nm. The relative permittivity of the biological dielectric (membrane proteins and lipids) is in the range of 4, while mineral barriers are efficient semiconductors [48,49,50,51] and therefore, strictly speaking, are not even dielectrics. For very short times, however, the finite velocity of charge movements may result in pseudo-dielectric behavior, although with apparent relative permittivities likely exceeding several thousand [52]. This implies that mineral membranes are unable to maintain electrostatic potentials over timescales exceeding microseconds [53] and therefore require permanent (and incommensurably higher than in chemiosmotic systems) recharge currents to preserve significant cross-barrier electrostatic potentials. This, in turn, would call for exceedingly high reactivities of the inner and outer sides of the mineral barriers to oxidize reductants and reduce oxidants, respectively. If despite all these hurdles, an electrostatic potential would build up over a mineral barrier, the resulting cation motive force would be three orders of magnitude weaker than in chemiosmosis due to the much shallower gradient over several μm rather than the 5 nm dielectric of the cytoplasmic membrane (F~∇U, see above). All the above indicates that mineral barriers cannot emulate the electrostatic characteristics of a chemiosmotic membrane in a living cell, a problem already raised previously [43,54].

Certain proponents of the alkaline vent theory consider the pH gradient as the fundamental source of free energy in mineral-walled pores of vent chimneys driving in particular CO_2_ reduction to organic building blocks [42,50,55,56,57]. In contrast to electrochemical gradients generated by metastable reductants and oxidants, which can mix in solutions while still maintaining the redox tension, pH gradients are due to concentration differences of protons in spatially separated compartments (Figure 1B). Apart from at the extremities of venting outlets, where new precipitation of mineral takes place (and certain macroscopic barriers within the vents, where local pH differentials span several tenths-of-a-millimeter-thick mineral precipitates [46]), we cannot see how any significant pH gradients should build up elsewhere in the microporous mound. Venturi effect-induced mixing of slightly acidic ocean waters into the alkaline vent fluid will necessarily result in steadily increasing pH values from the outer regions of the mound towards the inner venting channels, and individual pore-walls will, therefore, experience a continuum of pHs depending on their position within the mound but no sizable pH gradients across the walls [46]. Moreover, even if significant pH differences did exist as was observed with macroscopic mineral barriers in lab-reconstituted hydrothermal precipitates [46], the thickness of mineral barriers would again result in extremely shallow gradients yielding low ion-motive forces just as discussed for the electrostatic gradients above [43].

For all these reasons, it appears that thin-walled, low-permittivity organic vesicles are a decisive component for the emergence of the chemiosmotic mechanism [54].

### 1.4. Early Organic Vesicles Coating Mineral-Walled Micropores as the Primordial Chemiosmotic Capacitors

A host of different minerals associated in the past with alkaline vent chimneys have been shown to play a role in the synthesis of organic molecules, some of which subsequently can assemble into vesicles and liposomes [58,59,60,61,62]. Green rust has recently been demonstrated to promote the synthesis of a wide range of organics, including amphiphilic long carbon chains and amino acid precursors upon reacting with CH_4_ (methane oxidation) and CO_2_ (CO_2_ reduction), two redox species present in hydrothermal vent environments [63,64].

The ongoing synthesis of organic molecules within the hydrothermal mound will progressively coat the walls of its maze-like inner volume. Over time, most of the pore-like structures will become sealed and form topologically insulated vesicles with barrier dimensions in the range of a few nanometers and relative permittivities around 4 as typical for organic matter [65] (Figure 1C). Such vesicles would, therefore, feature the spherical-capacitor properties of chemiosmotic cells [66,67]. The reducing dissolved gases H_2_ and CH_4_ can diffuse through the barrier due to their charge neutrality while the soluble (negatively charged) oxidants such as nitrites and HCO_3_^−^ will remain excluded from the inner volume yielding a natural redox gradient, reducing inside and oxidizing outside, that is, precisely as in a bioenergetic membrane system. The weak oxidant CO_2_, even in small proportions compared to HCO_3_^−^ and CO_3_^2−^ when going towards the alkaline end of the pH gradient, can also reach the inner volume to there serve as feedstock for further organic syntheses (Figure 1C).

However, without further elements, the similarities to chemiosmosis end here. As mentioned above, the redox gradient in a chemiosmotic cell is converted into a transmembrane electrostatic potential which imparts a motive force on protons (and sometimes sodium cations) to migrate (via dedicated channels in the membrane) towards the inner volume, in the process, driving a displacement of phosphate to polyphosphate ratios away from equilibrium towards higher concentrations of polyphosphates. Because it lacks a transmembrane redox catalyst, the system is, at this point, unable to exploit the environmental redox gradient to power the electron and proton exchange across the organic vesicle dielectric membrane.

### 1.5. Green Rust May Bridge the Gap

The term «green rust» encompasses a group of naturally occurring or synthetic minerals usually occurring in the form of hexagonal crystals with dimensions in the hundreds of nanometers to µms. They are constituted of charged octahedrally-coordinated divalent Fe^2+/3+^ oxy-hydroxide sheets able to integrate other divalent metals (Ni, Co, Zn, Mg, Al) and separated by channel-like interlayers containing variable interchangeable anions (predominantly CO_3_^2−^, SO_4_^2−^, and Cl^−^ in order of interlayer affinity) which electrostatically stabilize the layers’ stacking [37,68,69]. Intriguingly, interlayer dimensions show significant plasticity upon incorporation of a wide range of molecules, from small anions to long-chain aliphatics [70,71], with interlayer heights that range from 7.9 Å to 40 Å, respectively, depending on the intercalating molecule [72].

Green rusts are notorious for promoting chemical catalyses mediated by the intercalation and adsorption of organics [70,71], as well as by redox reactivity towards redox partners (many of which likely present in AHV settings), most prominently the reduction of CO_2_ [64] and of NO_3_^−^ [73,74,75,76] and oxidation of CH_4_ [63], in addition to the reduction of organic compounds, a property frequently exploited in remediation processes (e.g., CT, TCE, TCM, chloroform) [77,78,79]. The versatile reactivity leads to the formation of a myriad of organic compounds, including amino acids and their precursors, as well as long carbon chain amphiphilic compounds. Such catalyses partly rely, in addition to surface reactivity, on the aforementioned intercalation of chemical precursors within the crystal’s layers where the reduced water activity is predicted to change the enthalpy–entropy landscape, decreasing the Gibbs free-energy, facilitating polymerization reactions [15,80]. Furthermore, green rust’s broad redox reactivity is supplemented by the high mobility of the exchanged electrons through the crystal via successive charge-hopping phenomena involving adjacent mixed-valence iron sites [53].

Relying on these well-established physico-chemical properties, nanocrystals of green rust have in the past been proposed to potentially fulfill the properties that would allow the above-mentioned organic vesicles in hydrothermal mounds to perform reactions akin to those of biological chemiosmosis [66,67,81,82,83]. In these scenarios, GR nanocrystals are considered to be inserted into the barriers of these organic vesicles and perform the required redox catalyses as well as bring about the electrostatic gradient-driven vectorial movements of electrons, protons, and polyphosphate anions [15,53]. While many (but not all) of these properties have been observed empirically on GR [63,73,74,84], the underlying assumption that GR can be inserted into hydrophobic barriers so far remains speculative. In this work, we probe the physico-chemical prerequisites for the conjectured integration of GR nanocrystals into proto-chemiosmotic membranes to actually occur.

## 2. Introduction

Determining AHV-relevant conditions that would have led to the formation of green rust crystals with such properties is a dual challenge given the usual hydrophilicity of the crystals which prevents their insertion in vesicles’ membranes and the numerous parameters influencing their very diverse sizes, which requires the assessment of several synthesis setups.

Firstly, green rust’s crystal size is influenced by many parameters, including growth time as well as metal composition and concentration [85,86,87]. Using X-ray diffraction (XRD) and transmission electron microscopy (TEM) analyses, green rust crystals were shown to range from tenths of nanometers to micrometers, with sizes increasing with growth time [86,87], and decreasing in the presence of nickel(II) [88,89]. In this paper, we focus on the effect of the presence of Ni(II) during green rust synthesis by co-precipitation. The decreasing size of green rust crystals under comparable conditions has already been reported previously [88,89]. In the emergence-of-life field, nickel-containing minerals have been recognized to mediate pertinent redox reactions and organic syntheses [14,56,57]. Even though nickel concentration in hadean/early archean AHVs was at least an order of magnitude lower than that of iron [35], its supply in large quantities is well described [15] and is associated with the presence of Ni-enriched minerals and geological formations during the Hadean [90,91,92]. The addition of Ni(II) in replacement for part of the Fe(II) in carbonate green rust syntheses has already been studied by XRD, showing a clear decrease in the crystal size in parallel to increasing amounts of Ni(II) [88,89].

Previous attempts to achieve hydrophobic functionalization strategies of LDHs and metal oxides were motivated by a wide range of objectives [93]. It improves the adsorption properties of minerals and favors their reactivity towards pollutants in remediation and in agronomical as well as industrial processes [94,95]. Modification of the structural or of the physico-chemical properties (i.e., hydrophobicity or delamination) [96,97] has also been reported, as well as of sensitivity to stimulus (i.e., light, electricity, magnetism) in suspension [98] or when embedded in organic vesicles for medical-related drug delivery [99,100,101,102,103,104]. To achieve these aims, a multitude of organic molecules, ranging from amphiphiles to polymers, have been used in the past. For this study, the focus was set on amino acids, given their interest in the emergence of life settings and non-disruptive behavior towards organic vesicles.

Amino acid interactions with LDHs and iron oxides have previously been studied for different purposes. For instance, a wide range of amino acids was tested by the Hibino group in order to modify the structure of minerals and, more specifically, to induce the delamination of Mg-Al LDHs [105,106]. Noteworthily, the effect of hydrophobic amino acids, especially tryptophan and phenylalanine, was found to differ from that of hydrophilic ones in that they did not induce delamination of the LDHs, apparently because their intercalation resulted in the formation of a hydrophobic sheet within the interlayer, preventing the separation of the LDH layers. In other cases, long-chain organics were used and were found to be efficient for delamination purposes [70,71]. Another example of the use of amino acids is to modify a mineral’s reactivity or its sensitivity towards other compounds [79], or alternatively to increase their biocompatibility [107].

The use of hydrophobic amino acids is still unusual, especially for the purpose of synthesizing hydrophobic particles, where amphiphilic aliphatics are often preferred. However, hydrophobic amino acids have several pertinent properties: (i) from an experimental point of view, they do not act as detergents against the liposomes we wish to incorporate the nanoparticle in, and (ii) from the point of view of emergence-of-life scenarios, they are likely to have been present in hydrothermal vent environments. Several groups have indeed shown that the synthesis of amino acids or precursors thereof could be catalyzed by green rust [108] or other iron-rich clay minerals under serpentinization conditions [109]. This makes the conditions for green rust functionalization by amino acids both more probable in the emergence of a life setting and more practical from an experimental perspective.

The framework for the study of amino acid interactions, adsorption, and polymerization on mineral surfaces is well-defined [110] and encompasses the experimental characterization methods, the sample states (gaseous, dried, or in solution), and the type of underlying interactions, which are also supported by computational dynamics [111,112].

The above-described experimental approaches served as starting points for our study and were refined to fit the known geochemical settings of early AHVs on the early Earth with the aim of working out the physico-chemical properties of green rust which would allow its functioning by fitting into the proto-chemiosmotic membrane setup as laid out above (Section 1.4). Given the high concentration of carbonates in the early AHV setup, our experiments focused on carbonate green rust as the one being predominantly precipitated in these geochemical conditions [33,113].

## 3. Material and Methods

### 3.1. Green Rust Syntheses

Carbonate green rust (GRCO_3_) syntheses were adapted from the method described by Barthélémy et al. [86] by co-precipitation upon mixing of an iron solution and a base solution corresponding to final concentrations of 0.133 M (mol·L^−1^) Fe(II)SO_4_•7H_2_O and 0.033 M Fe_2_(III)SO_4_•10H_2_O on one side, and 0.4 M NaOH and 0.233 M Na_2_CO_3_ on the other side. For nickel-GRCO_3_, 87% of the molar content in Fe(II) was replaced with Ni(II), corresponding to final concentrations of 0.017 M Fe(II)SO_4_•7H_2_O and 0.116 M Ni(II)SO_4_•7H_2_O. Additions of NaCl or MgCl_2_ were avoided to prevent the co-precipitation of chloride green rust at short growth times, as observed in our measurements by XRD.

For amino acid-functionalized green rust, the amino acid was added as a powder either in the base prior to green rust synthesis or in the green rust suspension afterward. At the end of the synthesis, the pH ranged between 11.5 and 12 and between 10.5 and 11 in the cases of unfunctionalized and functionalized green rusts, respectively.

### 3.2. Characterization of Green Rust Suspensions

Green rust suspensions were characterized at a range of time points after co-precipitation, especially during the first 24 h, using X-ray diffraction (XRD), scanning electron microscopy (SEM), and transmission electron microscopy (TEM). Green rust suspensions were centrifuged on a bench centrifuge within a glovebox under a N_2_/H_2_ atmosphere for 10 min at 14,000× *g* and then washed by retrieving the supernatant and replacing it with pure MilliQ water. These steps were repeated two times. For XRD characterization, the pellet paste retrieved after a third centrifugation step was then spread as a thin layer on zero-background Si wafers, dried using a desiccator, and inserted in a custom-built anoxic sample chamber equipped with a Kapton^R^ window. The sealed chamber was then removed from the glove box and XRD patterns were collected on an XPert Pro Panalytical™ diffractometer (Malvern Panalytical, Grovewood Road, UK) using Co Kα radiation in continuous scan mode with an equivalent 0.03° 2θ step, counting 3 h per sample over the 5–90° 2θ range. For electron microscopy imaging, the washing steps were followed by resuspension of the pellet in pure MilliQ water and by dilution to concentrations of 40 mM and 100 µM for SEM and TEM, respectively.

### 3.3. Point of Zero Charge (PZC) Determination Through pH Titrations

Point of zero charge (PZC) determinations were performed following the method developed by Guilbaud et al. [87] in 0.2 M green rust suspensions titrated with pure HCl (12 M) and 5 M KOH in µL step-wise additions and under continuous stirring inside a glovebox under N_2_/H_2_ atmosphere. A first titration was performed on a wide pH range (usually between pH 8 and 12) to obtain an approximate value for the PZC, followed by additional titrations over a narrower range of 1.2 to 1.5 pH units centered on the estimated PZC value, in order to avoid any phase dissolution or transformation due to steep pH shifts. The PZC value was determined graphically by taking the pH value at the intersection of the curve with the *x*-axis (i.e., pH value axis).

Alternatively, and to confirm the trends observed using this first method, a second method described by Angela et al. [107] was used. For the latter, 2 mL samples from the various GRCO_3_ syntheses were dried under N_2_ and resuspended in 8 mL of 0.1 M NaCl solutions at different pH values previously adjusted using KOH/HCl. The shift in pH upon resuspension of the GRCO_3_ pellets was monitored and plotted against each of the initial pH values.

### 3.4. SEM-XREDS (X-Ray Energy Dispersive Spectroscopy) Chemical Analysis

SEM-XREDS data were collected at the CINaM microscopy platform, using a JEOL JSM-7900F microscope equipped with a QUANTAX XFlash^®^ FlatQUAD annular four-channel silicon drift detector (Bruker, Billerica, MA, USA) for XREDS analysis. SEM imaging was performed at a tension of 5 kV at a working distance of 11 mm, and XREDS was performed to spatially detect N, Fe, and Ni in amino acid-functionalized green rust samples using the FlatQUAD detector through a PB-ZAF correction method. N, Na, S, Fe, Ni, Cu, and Al were all accounted for in the deconvolution and displayed in the atomic concentration calculation, while O and C were only accounted for in the deconvolution.

### 3.5. ATR-FTIR Spectra Acquisition

Fourier transform infrared (FTIR) spectra with a spectral resolution of 2 cm^−1^ were recorded using a VERTEX 70 (Bruker) spectrometer. A diamond attenuated total reflection (ATR) accessory (Quest, SPECAC, Orpington, UK) was used to study a 100 µL series of concentrated samples dried in a N_2_-flushed chamber. The samples consisted of GR syntheses among or after washing, or chloroform-resuspended green rusts from former pellets retrieved by centrifugation from the chloroform phase of hydrophobicity tests.

### 3.6. Hydrophobicity Tests and Hydrophobic Green Rust Retrieval

Degassed chloroform and GR suspensions were mixed in a 5 mL vial up to a total volume of 2 mL at 50/50 volume ratios. After allowing for phase separation, corresponding to an equilibration time of a few hours with short vortexing every hour, sedimentation of GR in the bottom chloroform phase was visually recorded, and the chloroform phase was retrieved using a syringe and centrifuged for 10 min at 14,000× *g*.

### 3.7. Raman Spectroscopy

Raman spectra were acquired using an inVia Reflex spectrometer (Renishaw, Wotton-under-Edge, UK) coupled to a THMS600 gas-tight cell (Linkam, Tadworth, UK) under 3 bar of argon on dry Ni(II)-Fe(II)-GRCO_3_ samples obtained by drying 200 µL of 0.2 M GRCO_3_ suspension on the cell crystal sample holder within a glovebox under N_2_ atmosphere. The cell was then closed within the glovebox to prevent any oxidation of the sample.

Spectra were acquired using a ×50 objective, a grating of 1200 lines/mm, and a monochromatic wavelength of 633 nm delivered by a HeNe laser at powers ranging from 5 to 10% of the 50 mW rated power for tryptophan-functionalized Ni(II)-Fe(II)-GRCO_3_, to 50% for unfunctionalized Ni(II)-Fe(II)-GRCO_3_.

### 3.8. X-Ray Photoelectron Spectroscopy

XPS spectra were acquired using a hemispheric electron analyzer (Resolve 120, PSP Vacuum, Orpington, UK) coupled to a non-monochromatized X-ray source (TX400, PSP Vacuum) comprising an Al Kα X-ray source operated at 140 W to prevent any radiation damage of tryptophan. The N1s spectra (400 eV) presented in this article have been recorded with a pass energy of 20 eV, a dwell of 10 s/point, and a step energy of 0.1 eV. The samples were prepared in the same conditions as for FTIR and Raman experiments. For tryptophan controls, tryptophan was dissolved at a 0.1 M concentration and pH was adjusted using KOH/HCl while for tryptophan-functionalized Ni(II)-Fe(II)-GRCO_3_ suspensions, the sample was washed during 4 cycles of centrifugation (10 min, 14,000× *g*), and resuspension in degassed water, to get rid of the excess sodium (Na).

### 3.9. Total Dissolved Iron Monitoring

Total iron concentration determinations were performed within a glovebox under a N_2_ atmosphere using a colorimetric method developed by Flinn Scientific (Batavia, IL, USA). Tested samples were obtained by retrieving the supernatant from different GRCO_3_ syntheses after centrifugation at 14,000× *g* for 10 min. In 96-well Tecan plates, 20 µL of sample or Fe(III)SO_4_ standards were mixed with 20 µL sodium-acetate buffer (1.2 M Na-acetate, 1.7 M acetic acid). A total of 10 µL of 140 mM hydroxylamine hydrochloride was added to oxidize all iron content, 10 µL of 18 mM 1–10 phenanthroline was then added to start the reaction, and the volume was adjusted to 200 µL using degassed MilliQ water (MilliporeSigma, Burlington, MA, USA). Absorbance was measured at 508 nm 15 min after 1–10 phenanthroline addition.

## 4. Results

### 4.1. XRD and Raman Characterization of GRCO_3_

**Figure 2:**
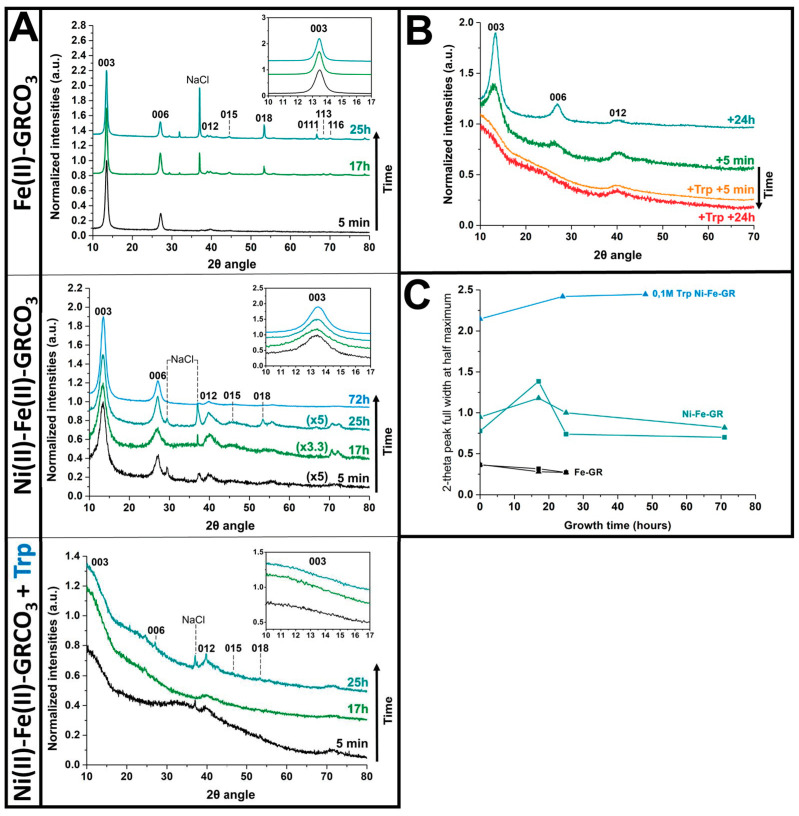
(**A**) X-ray diffractograms of Fe(II)-GRCO_3_ (top panel), Ni(II)-Fe(II)GRCO_3_ (middle panel), and 0.1 M tryptophan-functionalized Ni(II)-Fe(II)GRCO_3_ (bottom panel) as a function of time from 25 to 72 h. The inserts display a focus on the 003 peak to highlight its broadening due to nickel replacement and further tryptophan functionalization, as well as its progressive sharpening over growth time. (**B**) X-ray diffractograms over 24 h of Ni(II)-Fe(II)-GRCO_3_ with and without addition of 0.1 M tryptophan associated with TEM imaging Tryptophan addition at a 0.1 M concentration quickly leads to a strong broadening effect of all peaks. (**C**) Full width at half maximum for the 003 (triangles) and 006 (squares) peaks as a function of growth time for Fe(II)-GRCO_3_ (black), Ni(II)-Fe(II)-GRCO_3_ (dark green), and tryptophan-functionalized Ni(II)-Fe(II)-GRCO_3_ (light green) samples.

The effect of the partial substitution of Fe(II) by Ni(II) on the size of GRCO_3_ crystals was assessed by XRD, following the lead suggested by earlier work [88,89]. Peak broadenings of XRD patterns, as compared to the iron-only control crystals, indicate a substantial corresponding decrease in crystal size. Significant broadening without shifting of the peaks positioned at 7.51, 3.73, 2.65, 2.33, 1.96, 1.64, 1.58, and 1.55 Å corresponding to the (003), (006), (012), (015), (018), (0111), (110), and (113) green rust lattice planes, respectively [114], were observed (Figure 2A,C). This strong decrease in crystal size extended up to the maximum duration of 72 h of our measurements, during which the sample was observed to be stable, fully corroborating the data reported by Chaves and colleagues [88,89]. Additional functionalization of Ni(II)-Fe(II) GRCO_3_ by the addition of 0.1 M tryptophan following co-precipitation has a synergistic effect since it further dramatically intensifies the broadening of the green rust peak widths, yielding almost totally flattened diffraction peaks immediately after addition. The broadening of the main peaks (i.e., corresponding to the (003), (006), (012), and (018) green rust lattice planes) can be accompanied by a slight shift of the main peaks [115] while the less intense ones completely disappear from the diffractogram upon flattening.

The strongest broadening was attained when 0.1 M tryptophan was added 5 min after GRCO_3_ co-precipitation, while addition 24 h after co-precipitation or prior to co-precipitation (with in this case tryptophan directly dissolved in the base solution) resulted in weaker effects (Figure S1). For all timings of tryptophan addition, using tryptophan concentrations below 0.1M resulted in less pronounced broadenings (Figure S2). Adding higher final concentrations of tryptophan proved challenging due to solubility issues, even at the high pH values of our syntheses, which typically tend to increase the solubility of tryptophan. Adding 0.1 M tryptophan 5 min after the co-precipitation of Fe(II)-GRCO_3_ (without nickel) did not result in significant peak broadening as compared to its addition to Ni(II)-Fe(II)-GRCO_3_ suspensions. The kinetics of broadening in the absence of Ni(II) may have been too slow to be detected within the experimental timeframe (i.e., a few days). This indicates that the effect of tryptophan on green rust coherent diffracting domain size is conditioned by the presence of Ni(II) (Figure S3). Across all XRD kinetic analyses, peak broadening occurred simultaneously for all visible peaks without any significant changes in peak positions apart from some slight shifts. A potential effect of excess intercalating anions (in particular, Na_2_CO_3_) in preventing tryptophan intercalation on Ni(II)-Fe(II)-GRCO_3_ and Fe(II)-GRCO_3_ was probed via the removal of unintercalated Na_2_CO_3_ by washing after 72 h of growth and right before tryptophan addition. Removal of excess CO_3_^2−^ prior to the addition of tryptophan-influenced diffractograms only for the case of Fe(II)-GRCO_3_, resulting in the appearance of additional peaks under CO_3_^2−^ depletion as compared to control conditions. However, for the case of Ni(II)-Fe(II)-GRCO_3_, the removal of CO_3_^2−^ did not modify the effect of tryptophan addition, as evidenced by similar diffractograms (Figure S4).

The addition of 0.1 M tryptophan 5 min after co-precipitation of Ni(II)-Fe(II)-GRCO_3_ not only interrupts the usual peak-sharpening kinetics associated with the crystal growth of green rust but initiates an immediate and intense broadening of the peaks, lasting for up to several days (Figure 2B). Under these conditions (which will be used in the following experiments and referred to as “tryptophan-functionalized Ni(II)-Fe(II)-GRCO_3_)”, the characteristic XRD pattern of GRCO_3_ cannot be identified anymore by XRD, preventing unambiguous confirmation of persistence of green rust and indicating either an extreme decrease in crystal size or a complete loss of green rust during synthesis. Alternative experimental methods were, therefore, used to resolve this ambiguity (as will be detailed below).

Total dissolved iron was monitored as a function of growth time when tryptophan was present to check for potential durable dissolution of the green rust crystals induced by incubation with the amino acid (Figure S5). Four conditions were tested on both Fe(II)-GRCO_3_ and Ni(II)-Fe(II)-GRCO_3_ syntheses, i.e., the addition of 0.1 M tryptophan (a) 5 min after co-precipitation, (b) 72 h after co-precipitation or (c) 72 h after co-precipitation upon Na_2_CO_3_ depletion, and (d) without tryptophan addition. In Ni(II)-Fe(II)-GRCO_3_ syntheses, none of these conditions displayed significant dissolved iron content in the 0 to 72 h kinetics upon tryptophan addition. However, for Fe(II)-GRCO_3_ syntheses, the addition of 0.1 M tryptophan 5 min or 72 h after co-precipitation led to increased total dissolved iron concentrations ranging between 2 and 8 mM approximately, which, however, represents less than 5% of the initial iron input.

**Figure 3:**
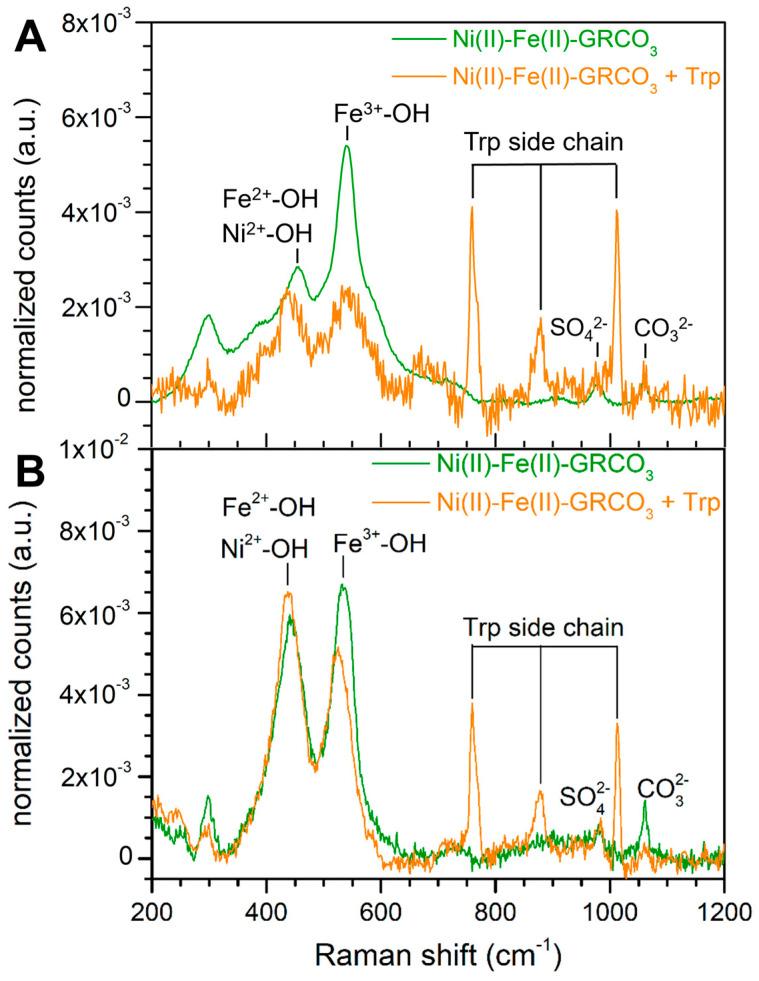
Raman spectra of solid (**A**) or in-solution (**B**) Ni(II)-Fe(II)-GRCO_3_ (green spectra) and tryptophan-functionalized Ni(II)-Fe(II)-GRCO_3_ (orange spectra) displaying GRCO_3_ and tryptophan vibrational bands in the 200–1200 cm^−1^ region and the decrease in the intensities ratio of the Fe^3+^-OH band and the Fe^2+^-OH/Ni^2+^-OH band upon tryptophan functionalization.

Raman spectroscopy has previously been used to characterize iron oxides, especially Fe(II)-GRCO_3_ and its oxidized forms [116,117]. Using this technique, we characterized Ni(II)-Fe(II)-GRCO_3_ and tryptophan-functionalized Ni(II)-Fe(II)-GRCO_3_ samples prepared similarly to those analyzed by XRD to verify whether GRCO_3_ crystals were still present under the conditions when the XRD diffractograms had broadened beyond detection. The spectra acquired on both Ni(II)-Fe(II)-GRCO_3_ and tryptophan-functionalized Ni(II)-Fe(II)-GRCO_3_ with tryptophan added 5 min after co-precipitation only display the pattern of vibrational bands corresponding to GRCO_3_, i.e., Fe(II)-OH/Ni(II)-OH at 455 cm^−1^, Fe(III)-OH at 540 cm^−1^, SO_4_^2−^ peak at 980 cm^−1^, and CO_3_^2−^ at 1060 cm^−1^ [116,117,118], confirming the presence of GRCO_3_ (Figure 3A,B). In addition, two bands arising from the indole function of tryptophan appear at 755 and 1010 cm^−1^ in the spectra of the tryptophan-functionalized Ni(II)-Fe(II)-GRCO_3_ sample (Figure 3B).

The intensity ratio of the Ni(II)-OH/Fe(II)-OH and the Fe(III)-OH bands varies between samples. The intensity of the Fe(III)-OH band is significantly larger than that of the Fe(II)-OH/Ni(II)-OH band in the Ni(II)-Fe(II)-GRCO_3_ spectra (Figure 3A) while their intensity appears equivalent in the tryptophan-functionalized Ni(II)-Fe(II)-GRCO_3_ sample (Figure 3B).

### 4.2. Electron Microscopy

Electron microscopy was used to confirm the effect of nickel replacement and tryptophan addition on the size, morphology, and structure of Fe(II)-GRCO_3_.

**Figure 4:**
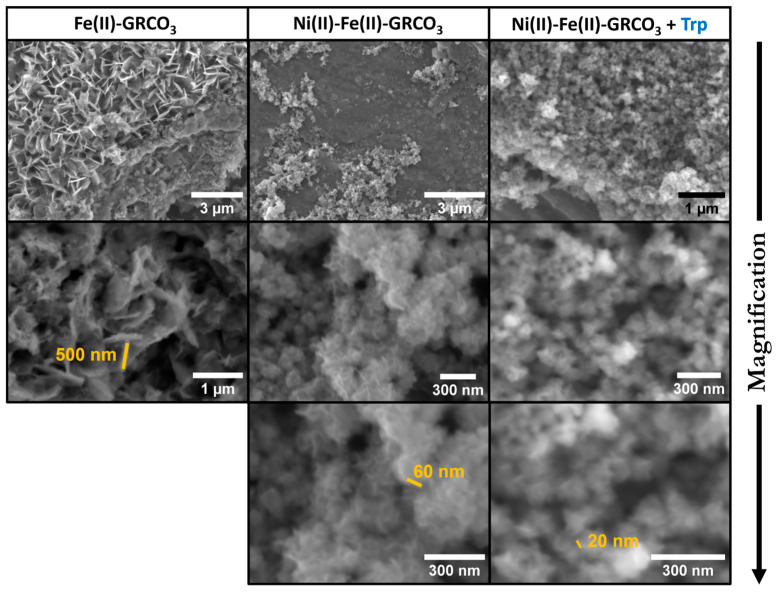
From left to right: SEM images of Fe(II)-GRCO_3_, Ni(II)-Fe(II)-GRCO_3_ and tryptophan-functionalized Ni(II)-Fe(II)-GRCO_3_, as observed in dropcasts 24 h after co-precipitation, from lower to higher magnifications going from top to bottom panels.

Figure 4 depicts SEM imaging performed 24 h after co-precipitation of the different (Ni(II))-Fe(II)-GRCO_3_ samples. These data support the trends observed in XRD: (i) replacement of 87% of the Fe(II) content by Ni(II) leads to a drastic decrease in crystal size from hundreds to tens of nanometers without noticeably altering their morphology and (ii) the addition of 0.1 M tryptophan 5 min after co-precipitation has a synergistic effect with Ni(II) by further reducing the size of the crystals, the largest of which rarely being wider than 20 to 30 nm, while crystals under 10 nm are probably undetectable using SEM.

**Figure 5:**
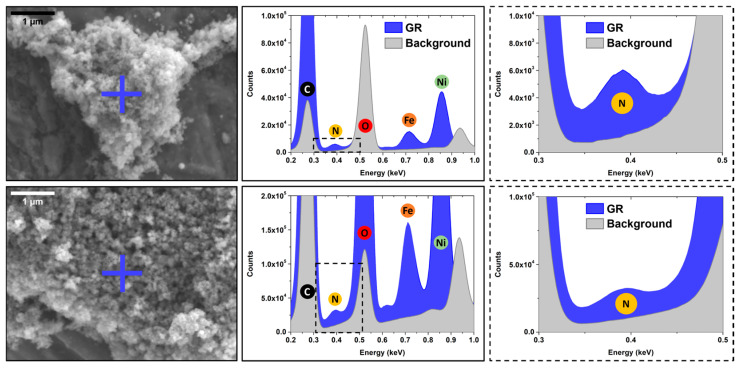
XREDS chemical analysis associated with SEM imaging, displaying spectra with levels of nitrogen (N) contained in tryptophan, and iron (Fe) and nickel (Ni) contained in green rust crystals during zone-specific observations of crystalline (blue and green spectra) and background (grey spectra) zones. (**Top**) Ni(II)-Fe(II)-GRCO_3_ functionalized with 0.1 M tryptophan added 5 min after co-precipitation, sampled 24 h after co-precipitation, with SEM image insert showing the respective sampled zones corresponding to the different spectra (matching cross and spectra). (**Bottom**) Ni(II)-Fe(II)-GRCO_3_ functionalized with 0.1 M tryptophan added 5 min after co-precipitation, sampled 5 min after co-precipitation, with SEM image insert showing the sampled crystalline zone (blue cross and spectrum).

Associated with SEM imaging, we performed XREDS chemical analyses to check for the spatial affinity of tryptophan and Ni(II)-Fe(II) GRCO_3_ through zone-specific detection of tryptophan-associated nitrogen (N) and Ni(II)-Fe(II)-GRCO_3_-associated iron and nickel (Fe, Ni). The zone-specific quantification was performed on both crystalline zones containing GRCO_3_ crystals and background zones devoid of crystals after washing the sample. The results show an increased content of nitrogen, nickel, and iron in the crystalline zones and a negligible presence of these atoms in the background, as expected due to the washing of the excess tryptophan from the GRCO_3_ pellets (Figure 5). This co-localization indicates a specific spatial interaction of tryptophan with Ni(II)-Fe(II) GRCO_3_ crystals.

**Figure 6:**
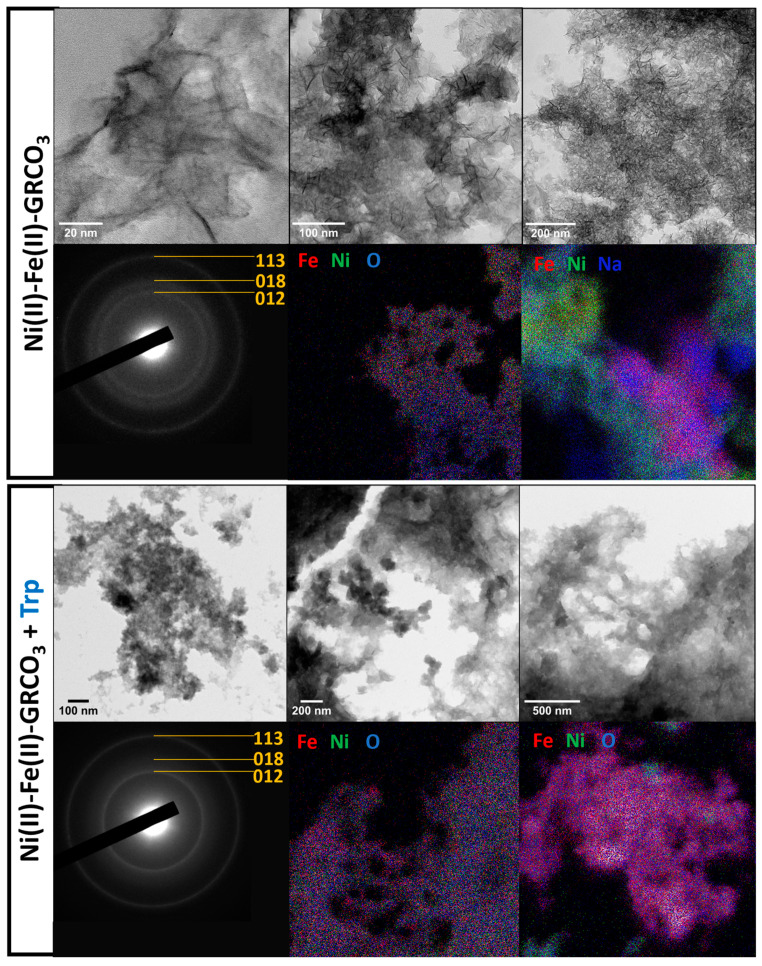
TEM images of Ni(II)-Fe(II)-GRCO_3_ (upper panels) and tryptophan-functionalized Ni(II)-Fe(II)-GRCO_3_ (bottom panels) as observed in dropcasts 24 h after co-precipitation, associated to electron diffractions and XREDS mapping. Imaging at higher magnifications clearly displays the synergistic effect of both Ni and tryptophan addition on the decrease of crystal size, while electron diffractions confirm that the observed crystals still correspond to green rust. Chemical mapping performed using XREDS confirms the co-localization of iron and nickel within the crystals, showing that they are co-precipitated within the same phase rather than independently, even in the presence of tryptophan.

TEM imaging (Figure 6) confirms the size decrease as well as the approximate mean crystal sizes determined by XRD and by the aforementioned electron microscopy techniques. It is of note that in the presence of tryptophan, the morphological outline of the crystals is more diffuse as compared to the sharper crystal edges of the control samples, due to coating by tryptophan and the thus-formed organic matrix. It is therefore more difficult to perform imaging at higher magnifications than on the non-functionalized Ni(II)-Fe(II)-GRCO_3_ samples. XREDS mapping targeting Fe, Ni, and O shows a high co-localization of these three atoms within the considered crystalline zones. However, punctual spatial heterogeneity is observed in the Fe/Ni distribution both in the presence and absence of tryptophan, with the appearance of phases containing either only nickel (green) or only iron (red) (Figure 6). Finally, and most importantly, electron diffraction experiments performed on crystal aggregates of both Ni(II)-Fe(II)-GRCO_3_ and tryptophan-functionalized Ni(II)-Fe(II)-GRCO_3_ both confirm the detection of three distances corresponding to the (113), (018) and (012) lattice planes of Fe(II)-GRCO_3_ or alternatively to the (100), (103) and (110) lattice planes of 5-line ferrihydrite.

### 4.3. Hydrophobicity Tests

**Figure 7:**
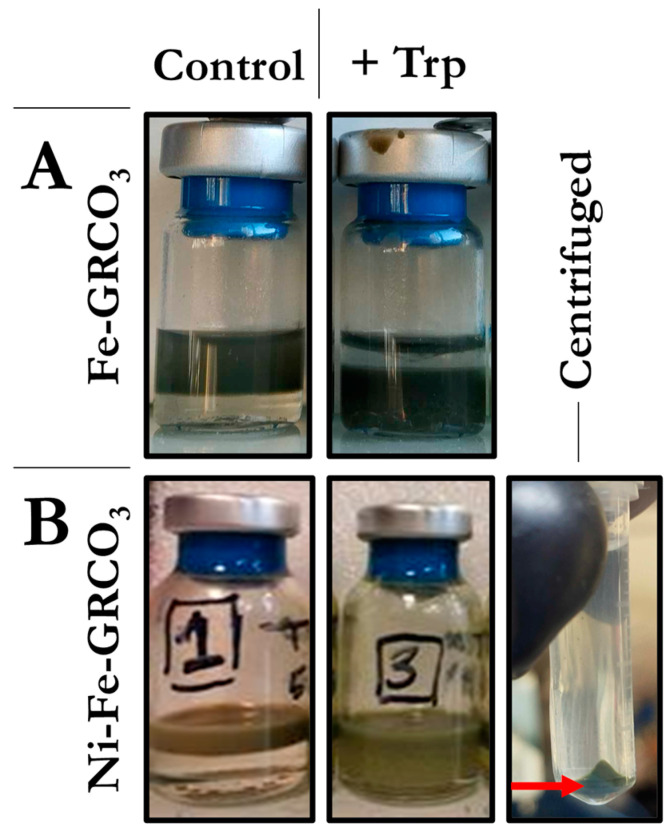
Hydrophobicity tests of GR samples added to a mixed phases solution of chloroform (bottom phase, hydrophobic) and water (upper phase, hydrophilic). (**A**) Hydrophobicity tests of standard Fe(II)-GRCO_3_ (**left**) or functionalized with 0.1 M tryptophan (**right**). (**B**) Hydrophobicity tests of Ni(II)-Fe(II)-GRCO_3_ (**left**) or tryptophan-functionalized Ni(II)-Fe(II)-GRCO_3_ (**right**) with the associated hydrophobic pellet obtained after centrifugation of the chloroform bottom layer (indicated by the red arrow).

Tryptophan is an amphiphilic amino acid that can interact both with the hydrophilic surface of green rust crystals and with hydrophobic surroundings. Hydrophobicity assays (as described in the Materials and Methods section) were performed on both standard Fe(II)-GRCO_3_ and Ni(II)-Fe(II)-GRCO_3_ prior to and after functionalization with 0.1 M tryptophan (added 5 min after co-precipitation, Figure 7) to determine the effect of tryptophan on the hydrophobicity of crystal surfaces. In both cases, no population of hydrophobic green rust crystals (i.e., green rust crystals partitioning into the bottom chloroform phase) was detected in the absence of tryptophan functionalization whereas the presence of tryptophan enabled a fraction of the sample to partition into the chloroform phase. The proportion of sample gaining hydrophobic character upon tryptophan functionalization appeared to be higher for standard Fe(II)-GRCO_3_ compared to Ni(II)-Fe(II)-GRCO_3_, resulting in a higher yield of mineral upon centrifugation-induced sedimentation of the chloroform phase.

### 4.4. Point of Zero Charge (PZC) Determinations

**Figure 8:**
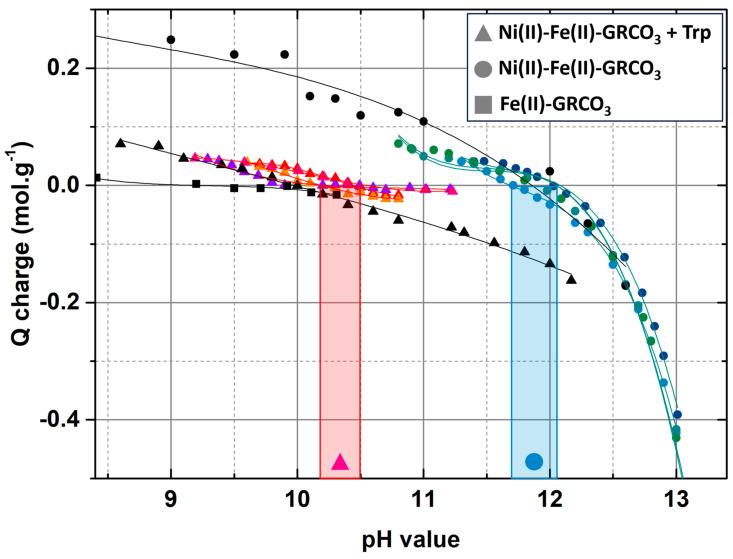
Point of zero charge (PZC) determination of various GR samples via pH titrations using the method described in Guilbaud et al. 2013 [87]. The PZC of Ni(II)-Fe(II)-GRCO_3_ (blue-green circles) decreases from approximately 11.8 (blue pH range) down to approximately 10.4 (red pH range) after functionalization with tryptophan (red-purple triangles), suggesting that tryptophan compensated part of the positive charges at the surface of the crystals.

Surface charge of Ni(II)-Fe(II)-GRCO_3_ and tryptophan-functionalized Ni(II)-Fe(II)-GRCO_3_ crystals were studied to determine the effect of tryptophan addition towards green rust’s surface charge and surface chemistry as well as potential electrostatic constraints applying to the crystal–amino acid interaction. The point of zero charge (PZC) value represents the pH at which the overall surface charge is neutral and it can be determined for different mineral species. Here, point of zero charge (PZC) determinations of the Ni(II)-Fe(II)-GRCO_3_ crystal surfaces were performed through pH titrations using the method developed by Guilbaud et al. [87] (Figure 8). The two titrations performed on Ni(II)-Fe(II)-GRCO_3_ samples (exhibiting a stable pH around 12) showed that the PZC of the crystals is in the range of pH 11.6 to 12 in the considered suspension (i.e., 0.233 M Na_2_CO_3_ and 0.4 M NaOH). Given that the stable pH of tryptophan-functionalized Ni(II)-Fe(II)-GRCO_3_ is between pH 10.8 and pH 11.2, that is, approximately one pH unit below the crystals’ PZC, the surface of the Ni(II)-Fe(II)-GRCO_3_ crystals is rather positively-charged upon addition of tryptophan. The same pH titrations were performed on tryptophan-functionalized Ni(II)-Fe(II)-GRCO_3_ suspensions, revealing a PZC between pH 10.1 and pH 10.4, approximately one pH unit below the PZC value of non-functionalized Ni(II)-Fe(II)-GRCO_3_ samples, indicating that a fraction of the crystals’ positive surface charges have become compensated. The trend of these observations was confirmed using the method described by Angela et al. for the PZC determination of magnetite [107] (Figure S6). The corresponding titrations indicate PZC values around pH 12 for unfunctionalized Ni(II)-Fe(II)-GRCO_3_ crystals, pH 11 for tryptophan-functionalized Ni(II)-Fe(II)-GRCO_3,_ and pH 10 for usual Fe(II)-GRCO_3_, supporting the results observed with the Guilbaud method.

### 4.5. Fourier Transform-InfraRed Spectroscopy Associated to X-Ray Photoelectron Spectroscopy

FTIR was performed to further investigate the type of interactions underlying the size and hydrophobic functionalization of Ni(II)-Fe(II)-GRCO_3_ by tryptophan. As described in the literature, pH significantly influences amino acid features in FTIR, primarily due to the protonation state of the amino and carboxylic groups as well as the side chains. Additionally, the protonation state of a specific functional group can indirectly affect the vibrational frequency of neighboring groups. Particularly, it is claimed that the protonation state of the amine function influences the vibrational state of the carboxylic acid function, explaining the shift of the peak corresponding to the latter when going from pH 6 to very alkaline pH, while the carboxylic acid is often already deprotonated above pH 3 [119,120].

**Figure 9:**
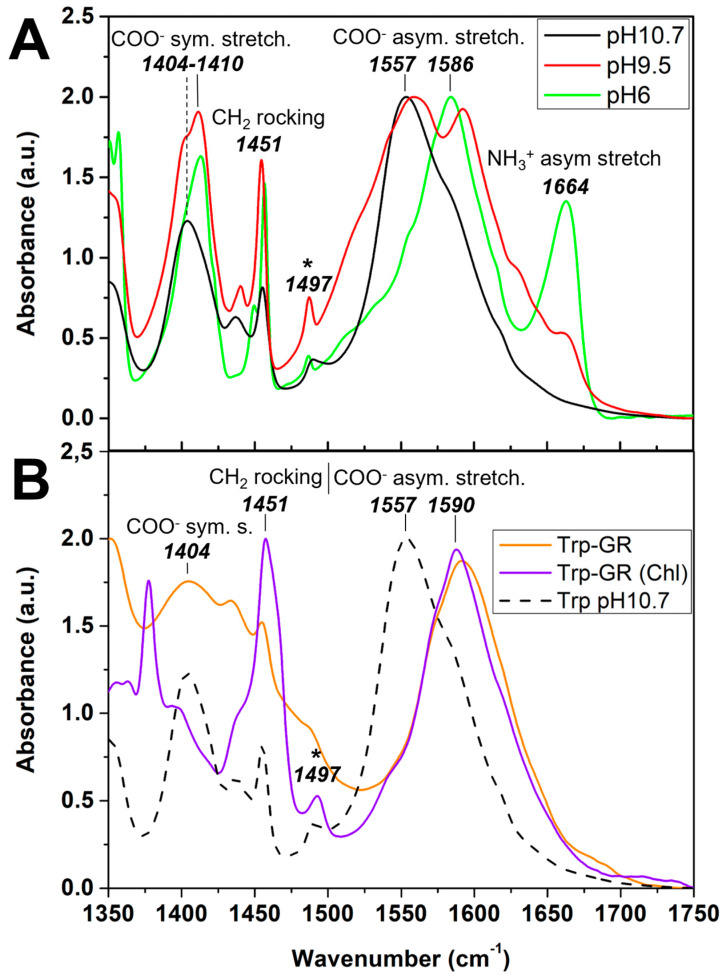
FTIR spectra of tryptophan in the 1350–1750 cm^−1^ region. (**A**) Tryptophan spectra at three different pH corresponding to its zwitterionic form (pH 6), anionic form (pH 10.7), and close to the amine function’s pKa (pH 9.5). (**B**) Comparison of the tryptophan spectrum at pH 10.7 (corresponding to the pH of tryptophan-functionalized Ni(II)-Fe(II)-GRCO_3_) with spectra of tryptophan during interaction with Ni(II)-Fe(II)-GRCO_3_, either after filtration of the excess tryptophan (green) or migration of hydrophobic green rust crystals into chloroform (orange). The peak corresponding to the C-C ring stretching of tryptophan, positioned at 1497 cm^−1^, is indicated by an asterisque “*”.

Here, spectra of tryptophan at five different pH values from pH 6 to pH 11.5 were recorded adjusting the pH with HCl/KOH. This allowed monitoring the modification of spectral features upon progressive deprotonation of tryptophan’s amine function, which converts from the zwitterionic (NH_3_^+^/COO^−^) to the anionic (NH_2_/COO^−^) form as the pH increases beyond the upper pKa value of the amine function (9.6). In zwitterionic tryptophan, the peak positioned at 1664 cm^−1^ is attributed to the asymmetric bending of the protonated amine function (NH_3_^+^) while the peak positioned around 1586 cm^−1^ is attributed to the asymmetric stretching of the deprotonated carboxylic acid function (COO^−^) (Figure 9A) as deduced from the experimental spectra of tryptophan [121,122], phenylalanine [123,124,125], and other amino acids [120,126,127,128,129,130,131,132]. Upon increase of pH, the peak attributed to the asymmetric bending of NH_3_^+^ in our tryptophan spectra disappears due to its progressive deprotonation to NH_2_. Numerous studies have demonstrated that the asymmetric stretching vibration of the deprotonated carboxylate group (COO^−^) in amino acids is sensitive to pH changes affecting the protonation state of the neighboring amine function. As the amine deprotonates along pH increase, the asymmetric stretching vibration of the COO^−^ function is claimed to shift down by 20 to 30 cm^−1^ [119,120,123,124,125,130,133].

In our case, at pH 6, this vibration typically appears around 1586 cm^−1^, but as the pH increases to 10.7, it shifts approximately 30 cm^−1^ downward to about 1557 cm^−1^. This shift is attributed to the deprotonation of the amino group and subsequent changes in the electronic environment surrounding the carboxylate moiety. The spectra recorded at pH 9.5, close the amino group’s pKa value of 9.6, feature both the 1557 cm^−1^ and the 1586 cm^−1^ peaks, an observation which we tentatively ascribe to the simultaneous presence of the two vibrational states of COO^−^ associated to populations of tryptophan with amine functions being either protonated (NH_3_^+^) or deprotonated (NH_2_) and thus exerting dissimilar influences on the vibrational frequencies of the COO^−^ function [120]. The further features present in the 1350–1750 cm^−1^ region of the spectra, including peaks at 1356 cm^−1^, 1408 cm^−1^, 1451 cm^−1^, and 1497 cm^−1^ are unambiguously attributed to CH bending, COO^−^ asymmetric stretching, CH_2_ rocking, and stretching of the benzene-pyrrole ring, respectively, in all of the aforementioned studies.

A spectrum of tryptophan was furthermore acquired during functionalization of Ni(II)-Fe(II)-GRCO_3_ by the amino acid at around pH 10.7 corresponding to the suspension’s pH upon tryptophan addition and after washing of the GRCO_3_ pellet to remove all non-interacting tryptophan molecules in the solution (Figure 9B). Removal of tryptophan limited to two washing steps was chosen to avoid significant pH drops due to excessive co-removal of NaOH and Na_2_CO_3_ in the suspension. The preparation of this sample for FTIR was the same as for SEM-XREDS (Figure 5), which showed that tryptophan molecules are specifically interacting with mineral phases, ensuring that the acquired spectra correspond to tryptophan molecules interacting with the crystal surfaces. As a control, the same sample was analyzed without washing to eliminate any potential pH effects from the washing procedure. The resulting spectra were identical, likely because the drying process concentrates tryptophan on the crystal surface, enhancing interactions and reducing the presence of non-interacting tryptophan molecules (Figure S7). Tryptophan-functionalized Ni(II)-Fe(II)-GRCO_3_ suspensions have pH values ranging from 10.8 to 11, which mainly corresponds to the anionic form of the amino acid. However, since the functionalization is efficient (according to hydrophobicity tests) at all pH values between 6 and 11, and that the amino acid can undergo protonation/deprotonation processes upon interaction with the mineral surface independently of the pH of the suspension, we used X-ray photoelectron spectroscopy (XPS) to determine tryptophan protonation state upon interaction with GR (Figure S8), using the same sample preparation as for FTIR and SEM-XREDS. XPS confirmed that tryptophan is in its anionic state during functionalization of GR, since only the deprotonated amine (NH_2_) and the indole’s NH peaks are detected in the N1s speciation of this sample. The spectra of tryptophan-functionalized Ni(II)-Fe(II)-GRCO_3_ displays one main peak positioned at 1591 cm^−1^ in the 1500–1700 cm^−1^ range, which we tentatively attributed to the asymmetric stretching peak of COO^−^, since no other feature is visible in this region in the control spectrum of anionic tryptophan at the same pH value of pH 10.7. This implies that the deprotonated carboxylic acid function (COO^−^) of tryptophan is involved in the interaction with the Ni(II)-Fe(II)-GRCO_3_ crystals surfaces upon functionalization, resulting in a 33 cm^−1^ peak-shift towards higher wavenumbers (i.e., lower vibrational frequencies).

Similar spectra recorded on tryptophan-functionalized Fe(II)-GRCO_3_ (i.e., not containing any nickel) (Figure S9) and on tryptophan associated with hydrophobic Ni(II)-Fe(II)-GRCO_3_ retrieved in chloroform upon tryptophan functionalization (Figure 9B) displayed the same features as the spectrum of tryptophan during functionalization of Ni(II)-Fe(II)-GRCO_3_ in suspension in water. This suggests that Ni(II) sites are not necessary for the interaction involving the tryptophan’s COO^−^.

Several other features vary in the different spectra: the overall broadening observed in the 1350 cm^−1^ to 1500 cm^−1^ region of the spectra of Trp-functionalized GR (dark green) corresponds to a stronger contribution of H_2_O vibrational frequencies, and can be explained by greater water retention of this sample during drying due to the presence of structural H_2_O within the GR crystals, while the hydrophobic GR pellet in chloroform is naturally devoid of H_2_O and the tryptophan control does not contain green rust, thus limiting the H_2_O-associated signals. The peak appearing at 1377 cm^−1^ in the spectrum of hydrophobic Trp-functionalized GR crystals in chloroform arises from chloroform itself.

## 5. Discussion

### 5.1. Ni(II) Substitution and Tryptophan Surface Adsorption Synergistically Result in Nanometric GR Crystals

XRD analyses show that the substitution of 87% of the Fe(II) content by Ni(II) in GRCO_3_ leads to a significant decrease in crystal size as evidenced by the intense peak broadening upon Ni(II) substitution, as already previously reported by Chaves et al. [88,89] (Figure 2A,C). Under these conditions, samples were found to be stable over several days. The addition of 0.1 M tryptophan 5 min after Fe(II)-Ni(II)-GRCO_3_ co-precipitation has a synergistic effect with Ni(II) substitution by intensifying the broadening phenomenon to the point that the characteristic GRCO_3_ peaks in the XRD diffractogram almost totally flatten and become difficult to discern (Figure 2A,C).

Diffractograms acquired for tryptophan addition at different timings (i.e., before co-precipitation or 24 h after co-precipitation) (Figure S1), at lower concentrations of tryptophan (Figure S2) and alternatively in standard Fe(II)-GRCO_3_ suspensions show that the most intense broadening corresponds to the condition under which tryptophan is added at a 0.1 M concentration 5 min after co-precipitation of Ni(II)-substituted Fe(II)-GRCO_3_, the only case in which peaks almost totally disappear. The synergistic effect of Ni(II)-substitution and tryptophan addition and the resulting intense flattening of peaks in the diffractogram entails the drawback that the resulting sample cannot be unambiguously identified by XRD anymore.

Such phenomenon could be due either to (i) a total dissolution of green rust in the presence of tryptophan, (ii) the transformation of green rust into an amorphous phase, or (iii) an extreme decrease in size yielding nanometric green rust crystals. The possibility that the observed extreme broadening of XRD features is due to the total dissolution of the green rust crystal following the addition of tryptophan without further re-precipitation of the dissolved iron content was ruled out by the quantification of total dissolved iron content which did not detect any measurable amounts of dissolved iron under these conditions (Figure S5).

However, iron resulting from dissolved green rust might have been mobilized into the formation of a mineral different from green rust following the addition of tryptophan, thereby preventing iron accumulation in the solution. To assess the presence of GRCO_3_ in the tryptophan-functionalized Ni(II)-Fe(II)-GRCO_3_ for which XRD characterization is impossible as outlined above, Raman spectroscopy was performed on both standard and tryptophan-functionalized samples. Raman spectra acquired on both samples display the same set of peaks attributed to the Fe(II)-OH/Ni(II)-OH and Fe(III)-OH functions of GRCO_3_, to excess SO_4_^2−^ and CO_3_^2−^ anions from the synthesis procedure, and to characteristic tryptophan vibrational modes for the case of tryptophan-functionalized samples (Figure 3). This result is complementary to the previous ones obtained in XRD by confirming that the extremely broadened XRD patterns observed after synergistic Ni(II) substitution and tryptophan functionalization still are entirely due to GRCO_3_ and therefore must reflect an extreme decrease in the size of individual crystals down to nanometric dimensions.

Further input from electron microscopy imaging provided a better picture of the impact of tryptophan addition on crystal morphology and corroborated the hypothesis of a decrease in size following the synergistic substitution of Ni(II) and the addition of tryptophan. Indeed, SEM imaging shows a clear size decrease upon Ni(II) substitution, with crystal sizes decreasing from hundreds to tens of nanometers, and the additional functionalization of tryptophan seems to even further reduce the size of the crystals, with dimensions never exceeding 20–30 nm (Figure 4). The associated EDX chemical analysis substantiates a spatial affinity between tryptophan and the Ni(II)-Fe(II)-GRCO_3_ crystals (Figure 5) through the co-localization of their constituting atoms, respectively, nitrogen on one hand and iron/nickel on the other. While XREDS is often used in a «mapping» mode to determine the chemistry of µm-sized single crystals [134] or macrostructures [135], the nanometric size of the crystals in our syntheses and the spot size constrain us to monitoring µm-sized crystalline zones containing a large number of crystals thus providing bulk-type information, as already explored in previous studies [136]. Measurements were performed using a FlatQUAD detector and 5 kV accelerating voltage of incident electrons, to facilitate the detection of light elements such as nitrogen (N). The energy spectra show a clear co-localization of N, Fe, and Ni content in the crystalline zones (blue and green spectra in Figure 5) containing Ni(II)-Fe(II)-GRCO_3_ crystals while background zones (grey spectra in Figure 5) devoid of crystals display negligible amounts of these atoms. This demonstrates that tryptophan molecules have a clear affinity for the crystals under these conditions. The atomic content ratio of nitrogen over iron and nickel in the crystalline zones was maximal 5 min after functionalization and decreases after 24 h, suggesting an optimal interaction at the beginning of tryptophan’s addition which is in line with its drastic effect at short times as seen in XRD (Figure 2B).

Finally, TEM imaging confirms the progressive size decrease phenomenon upon successive Ni(II)-substitution and tryptophan functionalization and corroborates via the associated XREDS analysis the close co-localization of Fe, Ni, and O atoms, suggesting the incorporation of Ni(II) mainly within the Ni(II)-Fe(II)-GRCO_3_ crystals and more rarely within Ni-only clusters (Figure 6). Most importantly, even if the morphological observation does not allow to unambiguously identify the presence of GRCO_3_, electron diffractions acquired during imaging allowed to retrieve three characteristic interatomic distances (2.6, 1.98 and 1.52 Å) corresponding to the (113), (018) and (012) lattice planes of GRCO_3_, or alternatively to the (100), (103) and (110) lattice planes of 5-line ferrihydrite. The presence of the latter was, however, ruled out by correlation with Raman spectroscopy (Figure 3). The detection of these diffraction patterns which were invisible in X-ray diffraction is a usual phenomenon due to the short wavelength of electrons (0.025 Å at 200 kV), which occurs when dealing with a very small coherent crystalline domain [137,138]. This analysis confirms the hypothesis of the formation of nanometer-sized tryptophan-functionalized crystals of Ni(II)-Fe(II)-GRCO_3_ by the synergistic effect of Ni(II)-substitution and tryptophan addition on the decrease of GRCO_3_ crystal size.

Such a decrease in the crystals’ dimensions upon tryptophan addition could be explained by intercalation-related delamination processes. While the broadening of the peaks in our XRD experiments can be accompanied by a slight shift of the peak position [115], the absence of a significant shift of the peak corresponding to the interlayer spacing as well as the simultaneous broadening of all peaks in all experiments suggests that there is no delamination-related broadening process provoked by potential intercalation of tryptophan in the interlayers. This conclusion is supported by previously reported observations on a similar LDH and rationalized by the hypothesis that the hydrophobic character of tryptophan prevents delamination upon intercalation due to the formation of a strongly hydrophobic region corresponding to the interaction of tryptophan’s side chains in the interlayer [106].

However, the theoretically postulated intercalation-related peak shift [106] is not observed in our experiments, which may be due to the high concentration of intercalating anion in our samples (i.e., 0.233 M of CO_3_^2−^) exceeding the concentration of tryptophan and thus preventing its access to the interlayer by competition [105]. To assess this hypothesis, XRD was performed on tryptophan-functionalized Ni(II)-Fe(II)-GRCO_3_ and Fe(II)-GRCO_3_ prior to and after removal of the excess CO_3_^2−^ in solution by washing (Figure S4). Only in the case of Fe(II)-GRCO_3_ did the removal of CO_3_^2−^ lead to severe phase transformations upon tryptophan addition, suggesting that the presence of highly concentrated CO_3_^2−^ prevents tryptophan intercalation in the absence of Ni(II), while in Ni(II)-Fe(II)-GRCO_3_, the tryptophan does not intercalate no matter the amount of excess CO_3_^2−^.

Taken together, these results suggest that the synergistic interaction of tryptophan with Ni(II)-substituted Fe(II)-GRCO_3_ leads to a drastic size decrease of the crystals and is associated with surface adsorption rather than intercalation of the amino acid. The intercalation/delamination hypothesis being ruled out, it cannot justify such size decrease kinetics. By default, and accounting for the iron/nickel heterogeneities observed in TEM/XREDS, we propose that the effect of tryptophan could be due to the dynamic dissolution–precipitation phenomenon ongoing in Ni(II)-Fe(II)-GRCO_3_ suspension throughout growth time. In the first minutes after co-precipitation, Fe(II) or Ni(II)-rich phases are precipitated and quickly dissolved to serve the more thermodynamically stable nucleation of new Fe(II)-rich or Ni(II)-rich phases in a dynamic and continuous manner, explaining the appearance of iron-only and nickel-only crystals in our TEM/XREDS observations. In the presence of tryptophan, the growth of these newly formed nuclei could be severely slowed down, due to tryptophan coating the crystals and competing for crystallization sites. Progressively, larger crystals would dissolve and precipitate into new nuclei, the size of which is limited to a few nanometers in the presence of tryptophan, thus explaining the progressive reduction in the size of the crystals in suspension.

### 5.2. Generation of Hydrophobic GR Crystals by Functionalization with Tryptophan

To assess whether the aforementioned surface functionalization of GRCO_3_ by tryptophan, an amphiphilic amino acid that potentially can simultaneously interact with the hydrophilic surface of green rust and with a hydrophobic environment, allows to obtain hydrophobic green rust crystals, hydrophobicity tests were performed. The functionalization by tryptophan of both Fe(II)-GRCO_3_ and Ni(II)-Fe(II)-GRCO_3_ led to the recovery of part of the samples in the bottom chloroform phase reflecting the crystals’ hydrophobic nature, while unfunctionalized samples remained in the upper aqueous phase (Figure 8).

The specifics of the interaction between tryptophan and the surface of Ni(II)-Fe(II)-GRCO_3_ yielding hydrophobic crystals were determined using a combination of Fourier transform infrared spectroscopy (FTIR) characterization, and point of zero charge (PZC) determination. The ensemble of these approaches allowed us to identify the chemical functions involved in the interaction and to decipher their effect on the crystal’s surface charge, respectively.

FTIR was performed to pinpoint the chemical functions on the tryptophan molecule which engage in interaction with green rust crystals’ surfaces during adsorption. Upon Ni(II)-Fe(II)-GRCO_3_ functionalization, the spectrum of tryptophan undergoes significant changes compared to the control spectra acquired at the same pH (Figure 9). Under these conditions, only a single peak is observed in the 1500–1700 cm^−1^ region (positioned at 1590 cm^−1^), a pattern exclusively found in control spectra (recorded in the range of pH 6 to pH 11.5) when the tryptophan is in its anionic state (i.e., at high pH-values). Although the pH of the tryptophan-functionalized Ni(II)-Fe(II)-GRCO_3_ suspension is at 10.7 and thus corresponds to pH-values where tryptophan alone is in its anionic form (displaying a single peak due to asymmetric COO^−^ stretching at 1557 cm^−1^), tryptophan’s interaction with the crystal surfaces may well shift the relevant pk-values of its functional groups.

Moreover, our hydrophobicity assays showed that tryptophan-functionalization of Ni(II)-Fe(II)-GRCO_3_ efficiently produced hydrophobic crystals at both pH 6 and pH 10.7 further motivating the necessity to check whether tryptophan remained in its anionic form upon functionalization, as the adsorption process can locally modify tryptophan’s protonation state independently of the pH value. To determine the in-situ protonation state of tryptophan during interaction with Ni(II)-Fe(II)-GRCO_3_, we performed XPS analyses on samples of tryptophan-functionalized Ni(II)-Fe(II)-GRCO_3_ prepared under the same conditions as for FTIR and the previous techniques (Figure S6). The identification of the deprotonated amine function (NH_2_) via this approach confirms that the tryptophan is in its anionic form in these samples. Furthermore, the EDX analysis performed previously on the same sample under similar conditions confirms that the tryptophan spectra recorded in FTIR very strongly resemble those of tryptophan molecules interacting with Ni(II)-Fe(II)-GRCO_3_ crystals.

Summarizing this information, we suggest that the isolated 1590 cm^−1^ peak detected in the spectra of anionic tryptophan upon functionalization of Ni(II)-Fe(II)-GRCO_3_ may correspond to the isolated COO^−^ asymmetric stretching peak positioned at 1557 cm^−1^ in the control spectra of the anionic tryptophan recorded at the same pH. The same 1590 cm^−1^ peak is present in spectra recorded on centrifugation pellets containing hydrophobic tryptophan-functionalized Ni(II)-Fe(II)-GRCO_3_ crystals that had partitioned into the chloroform phase during hydrophobicity assays (Figure 7). Again, this band is attributed to the asymmetric COO^−^ stretching mode. Interaction of tryptophan with the Ni(II)-Fe(II)-GRCO_3_ crystals (conferring hydrophobic characteristics to these crystals), thus induces a shift of the COO^−^ asymmetric stretching peak from 1557 cm^−1^ to 1590 cm^−1^ in the FTIR spectra. According to previous studies, such a shift is routinely observed during interactions of amino acids or carboxylic acids with LDH mineral surfaces or iron oxides and is attributed to hydrogen bonding or electrostatic interactions [128,132,135,139,140].

The structural details of these interactions, that is, representing either a monodentate binding mode (i.e., involving either of the two oxygens of the COO^−^ functional group) or a bidentate one (i.e., involving both oxygens), depend on the spacing between the peaks arising from the symmetric and the asymmetric stretching mode of COO^−^. A separation of 150 cm^−1^ is considered to rather indicate a monodentate type of interaction while higher spacings (towards 250 cm^−1^) would correspond to bidentate types of interaction [141]. In our experiments, this spacing increases from 153 cm^−1^ in the tryptophan control at pH 10.9 to 186 cm^−1^ in the sample in which tryptophan interacts with Ni(II)-Fe(II)-GRCO_3_, suggesting a weak bidentate mode of interaction. The observed 33 cm^−1^ shift can be converted into a bonding energy for the COO^−^ function of approximately 46 kJ.mol^−1^ [142]. This bonding energy value, added to the fact that a layer of partly-protonated oxygen bridges cover the Ni and Fe sites in GRCO_3_ surface structure, as well as computational and experimental studies on amino acids and carbonate interactions with other LDH [111,143,144] suggests that tryptophan’s COO^−^ are establishing bidentate hydrogen bonds with GRCO_3_ surface hydroxides.

The possibility of tryptophan engaging in an electrostatic interaction of the hydrogen-bonding type with the surface of GRCO_3_ crystals through its negatively charged deprotonated COO^−^ functional group is mainly conditioned by the pH-dependent protonation state of the oxygen bridges and the resulting overall surface charge of the crystals. However, local interactions could still occur independently of these conditions.

To check for possible electrostatic interactions and the effect of tryptophan adsorption on crystals’ surface charges, we performed point of zero charge (PZC) determinations through pH titrations. Our titrations reveal a PZC value of non-functionalized Ni(II)-Fe(II)-GRCO_3_ crystals of approximately pH 12, making the crystal’s surface rather protonated and positively charged at the pH value of 10.7 obtained in the tryptophan-functionalized Ni(II)-Fe(II)-GRCO_3_ suspension (Figure 9). This would then favor the presumed hydrogen bond-type of electrostatic interaction observed in FTIR involving tryptophan’s negatively-charged COO^−^ function with a protonated positively-charged surface of Ni(II)-Fe(II)-GRCO_3_ crystals. Upon tryptophan functionalization, the PZC value of the crystals in suspension decreases down to approximately pH 10.4, which can be interpreted as tryptophan molecules neutralizing part of the positive charges at the surface of Ni(II)-Fe(II)-GRCO_3_ along their electrostatic interaction, thus artificially decreasing the PZC value of the crystals. This trend of PZC decrease upon tryptophan adsorption (Figure 8), confirmed using an alternative method (Figure S6), supports the hypothesis of hydrogen bonding involving tryptophan’s COO^−^ sites and Ni(II)-Fe(II)-GRCO_3_ hydroxide sites according to our FTIR experiments.

### 5.3. Implications for the Formation of AHV-Hosted Proto-Chemiosmotic Membranes

The combination of the green rust mineral with both nickel and tryptophan, two likely compounds present in the vicinity of AHV during the archean/hadean era, arising from geochemical processes or direct redox-mediated mineral catalysis, respectively, turns these minerals into nanometric and hydrophobic redox catalysts. The impetus for assessing the plausibility of these modifications in green rust crystals’ properties comes out of the acknowledgment that the mineral membranes in AHV mounds cannot straightforwardly harbor chemiosmotic processes. Indeed, their thicknesses, permittivities, and conductivities vastly exceed those of biological dielectric barriers and therefore are unsuited for the maintenance of pH- and electrostatic gradients as would be required for a chemiosmotic system. A solution to this problem can be proposed by positing that sealed organic barriers coating the walls of micropores in the mound rather than the mineral barriers themselves have provided the environment for chemiosmosis to emerge. In this scenario, redox-mediated organic syntheses catalyzed by green rust and fueled by redox compounds available in the AHV environment (i.e., CH_4_, CO_2_) would have produced long carbon-chain amphiphiles and amino acid precursors leading to the formation of the mentioned sealed organic vesicle. Our study demonstrates that green rust can indeed feature the physico-chemical properties required for its incorporation in these organic membranes in the presence of AHV-hosted/produced nickel and tryptophan. In such a system, green rust may couple the transfer of electrons from electron donors, having diffused towards the inner volume (H_2_ and CH_4_) to the acceptors retained outside (HCO_3_^−^ and NO_3_^−^) to the build-up of ∆Ψ in ∆pH across the vesicles’ membranes.

Our future research will focus on the experimental setting up of such a system and on measuring its functional parameters. For this purpose, we will aim to develop a protocol for incorporating hydrophobic and nanometric green rust crystals into liposome membranes. This approach will rely on established methods of incorporation of nanoparticles in vesicles’ membranes, modifying them to suit our experimental setup, while optimizing the lipid composition. Successfully incorporating green rust crystals into liposome membranes would provide the first experimental validation of the scenario put forward in this article: the formation of a protocell embedding green rust nanocrystals.

As a next step, such systems will be used to decipher the ability of green rust to perform the coupled transfer of protons and electrons across the liposome membranes. Upon application of a transmembrane potential (∆Ψ) and monitoring the resulting ∆pH, two parameters that can be measured with the help of fluorescent probes such as oxonol IV or HPTS, respectively, we will try to quantify the extent and kinetics of these processes.

These suites of experiments are intended to assess whether green rust may, at life’s emergence, have been able to perform chemiosmosis-related reactions that are carried out by metalloenzymes in extant life.

## 6. Conclusions

In this work, we demonstrate that specific environmental parameters likely to prevail in hadean/archean AHVs, i.e., the presence of nickel and amino acids (here exemplified by tryptophan), influence the characteristics of green rust crystals in a way relevant to their incorporation into the membranes of organic vesicles.

We specifically explore the changes in both the size and hydrophobicity of green rust crystals, two critical parameters for their incorporation into organic membranes. Based on previous works on green rust’s crystallization, growth and interlayer and surface chemistry, we developed a set of analytical techniques to characterize these modifications, including the following: (i) X-ray diffraction (XRD) to assess the structure and size decrease of green rust upon exposure to nickel and amino acids, (ii) Raman spectroscopy, to confirm the presence of green rust upon tryptophan addition when the XRD signal was not informative, (iii) transmission and scanning electron microscopy (TEM, SEM) to analyse their size and morphology, in addition to their structure using electron diffraction and chemical composition using XREDS to localize tryptophan at the surface of the crystals, (iv) hydrophobicity tests to evaluate the hydrophobic properties of the crystals upon tryptophan surface functionalization, (v) point of zero charge (PZC) of the crystals to determine the effect of tryptophan adsorption on surface charge and the associated interaction modes, and (vi) Fourier transform infrared spectroscopy (FTIR) to identify the chemical functions involved in the surface functionalization by tryptophan and deduce the type of interaction underlying the phenomenon.

Firstly, the synergistic effect of the combined presence of Ni(II) and tryptophan yields nanometric carbonate green rust crystals as soon as they co-precipitate without altering their basic structure or stability. This drastic decrease in size enables green rust crystals to reach the thickness range of organic membranes. Secondly, the electrostatic adsorption of tryptophan to these crystals renders their surfaces hydrophobic, thus matching the hydrophobic core of the aforementioned membranes and favoring their insertion into these membranes. Our multi-parameter experimental study demonstrates that green rust crystals, under the effect of environmental parameters compatible with those of AHVs, may be able to integrate the barriers of organic vesicles. These minerals, capable of reducing CO_2_ and oxidizing CH_4_, may then have acted as redox catalysts in a proto-chemiosmotic system. Furthermore, in such settings, green rust may have played the role of the inorganic precursor of current chemiosmotic metalloenzymes, transferring electrons across the vesicle’s membranes following the redox gradient, while simultaneously transporting protons, ultimately forming a transmembrane ∆pH. To further evaluate the hypothesis, the next step involves integrating green rust nanocrystals synthesized according to the protocols developed in this work into artificial vesicles (liposomes). Within this vesicle, we will measure green rust-mediated electron and proton transport as well as the build-up of electrostatic gradients. Experiments towards these objectives are currently underway in the research teams involved in this study.

## Data Availability

The raw data supporting the conclusions of this article will be made available by the authors on request.

## Supplementary Materials

The following supporting information can be downloaded at https://www.mdpi.com/article/10.3390/life15040671/s1, Figure S1: X-ray diffraction of Ni(II)-Fe(II)-GRCO_3_ functionalized with tryptophan by addition in the base before co-precipitation (top panel), or in the suspension 5 min (middle panel) or 24 h (bottom panel) after co-precipitation. Figure S2: X-ray diffraction of Ni(II)-Fe(II)-GRCO_3_ functionalized with 0.1 M (top panel) or 0.05 M (bottom panel) tryptophan by addition 5 min after co-precipitation. Figure S3: X-ray diffraction of Fe(II)-GRCO_3_ functionalized with 0.1 M tryptophan by addition 5 min after co-precipitation. Figure S4: Cu-source X-ray diffraction of Fe(II)-GRCO_3_ (A) or Ni(II)-Fe(II)-GRCO_3_ (B) functionalized with 0.1 M tryptophan by addition after 72 h growth with (red spectra) or without prior removal of excess CO_3_^2−^ by washing. Figure S5: Monitoring of total dissolved iron of both Fe(II)-GRCO_3_ (black) and Ni(II)-Fe(II)-GRCO_3_ (green) samples at 0 h, 24 h, 48 h, and 72 h after 0.1 M addition either 5 min after co-precipitation (filled squares), 72 h after co-precipitation (filled up-triangles) or 72 h after co-precipitation upon Na_2_CO_3_ removal (filled down-triangles) and without tryptophan addition (control, filled circles). Figure S6: Point of zero charge (PZC) determination of various GRCO_3_ samples via resuspensions of dry pellets of GRCO_3_ using the method described in Angela et al. Figure S7: FT-infrared spectra of tryptophan in the 1350–1750 cm^−1^ region during interaction with Ni(II)-Fe(II)-GRCO_3_ with (green spectrum) or without (red spectrum) washing of the excess tryptophan in solution prior to drying, and compared to the control spectrum of anionic tryptophan (black dashed spectrum). Figure S8: X-ray photoelectron spectroscopy (XPS) speciation of tryptophan-associated nitrogen in control zwitterionic (bottom spectrum) and anionic (middle spectrum) tryptophan compared to tryptophan interacting with Ni(II)-Fe(II)-GRCO_3_ (top spectrum), displaying the protonated state of tryptophan’s amine function in these conditions. Figure S9: FTIR spectra of tryptophan (black) in the 1350–1750 cm^−1^ region during interaction with Ni(II)-Fe(II)-GRCO_3_ (purple) or Fe(II)-GRCO_3_ (orange) upon retrieval of corresponding functionalized suspensions in chloroform during hydrophobicity tests.

## Abbreviations

The following abbreviations are used in this manuscript:

AHV	Alkaline hydrothermal vents
LDH	Layered double-hydroxide
GR	Green rust
XRD	X-ray diffraction
SEM	Scanning electron microscopy
TEM	Transmission electron microscopy
PZC	Point of zero charge
XREDS	X-ray energy dispersive spectroscopy
ATR-FTIR	Attenuated total reflection-Fourier transform tnfraRed spectroscopy
XPS	X-ray photoelectron spectroscopy
Trp	Tryptophan
Chl	Chloroform
