# Supplementary material for: Nanometric and Hydrophobic Green Rust Minerals upon Exposure to Amino Acids and Nickel as Prerequisites for a Primitive Chemiosmosis
Source: Life (Basel). 2025 Apr 19;15(4):671. doi: 10.3390/life15040671 (PMC12028411; doi:10.3390/life15040671)
Supplement: Supplementary file 1 [file life-15-00671-s001.zip › life-3544951-supplementary.pdf]

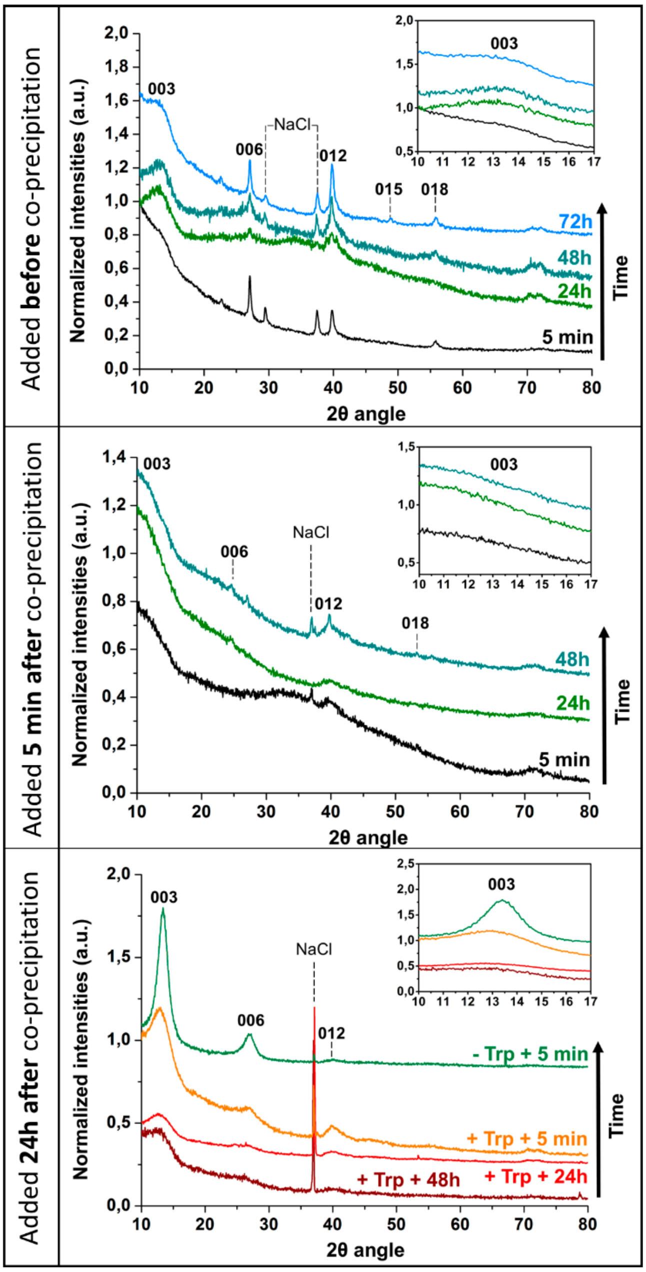

Supplementary information Figure S1 : X-ray diffraction of Ni(II)-Fe(II)-GRCO$_3$ functionalized with tryptophan by addition in the base before co-precipitation (top panel), or in the suspension 5 minutes (middle panel) or 24 hours (bottom panel) after co-precipitation. Inserts focus on the 003 peak to depict its broadening due to nickel replacement and further tryptophan functionalization, as well as its progressive sharpening over growth time.

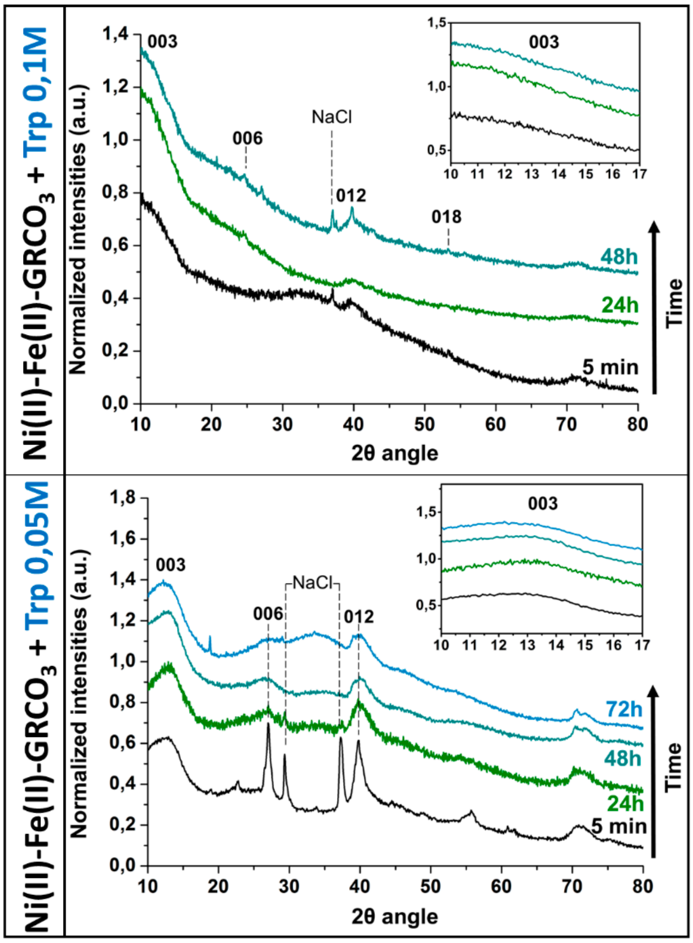

Supplementary information Figure S2: X-ray diffraction of Ni(II)-Fe(II)-GRCO₃ functionalized with 0.1 M (top panel) or 0.05 M (bottom panel) tryptophan by addition 5 minutes after co-precipitation. Inserts focus on the 003 peak to highlight the broadening due to nickel replacement and further tryptophan functionalization, as well as its progressive sharpening over growth time.

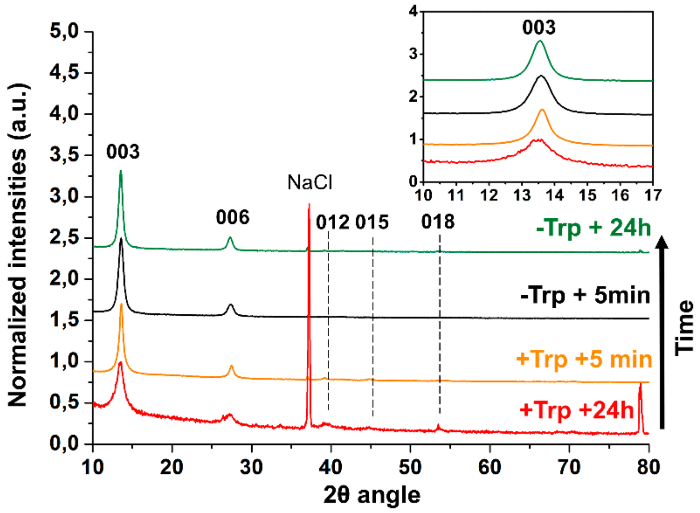

Supplementary information Figure S3: X-ray diffraction of Fe(II)-GRCO₃ functionalized with 0.1 M tryptophan by addition 5 minutes after co-precipitation. Inserts focus on the 003 peak to highlight the broadening due to nickel replacement and further tryptophan functionalization, as well as its progressive sharpening over growth time.

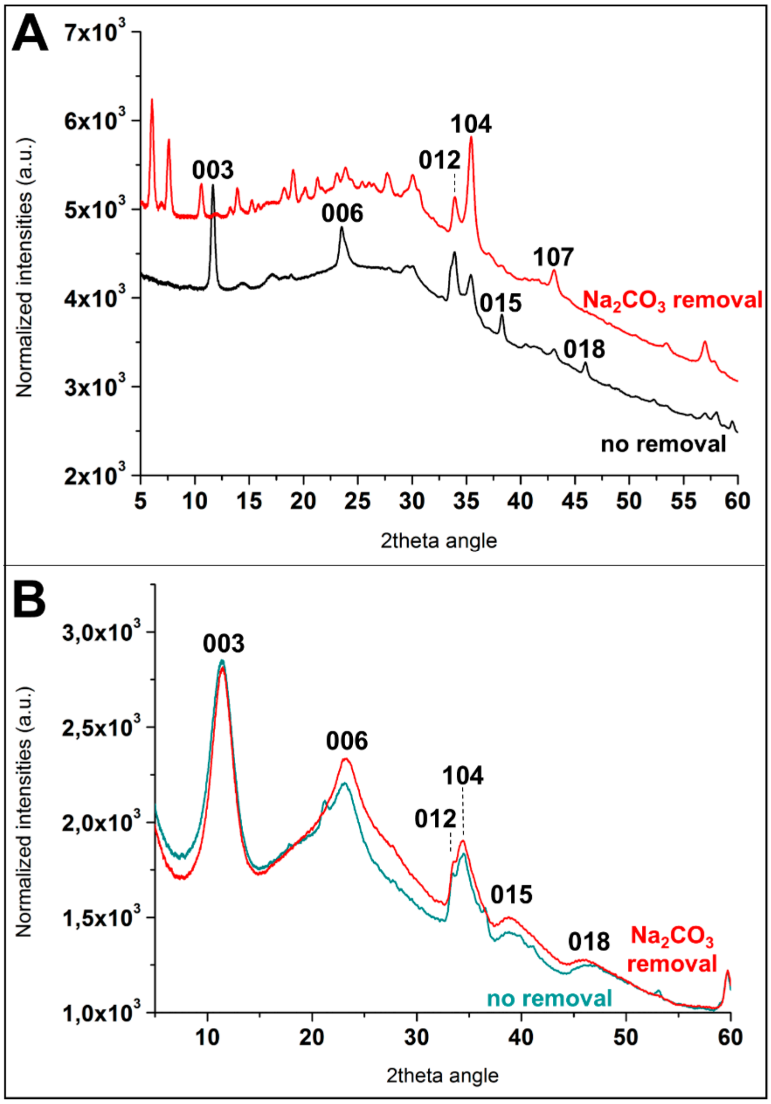

Supplementary information Figure S4 : Cu-source X-ray diffraction of Fe(II)-GRCO$_3$ (A) or Ni(II)-Fe(II)-GRCO$_3$ (B) functionalized with 0.1 M tryptophan by addition after 72h growth with (red spectra) or without prior removal of excess CO_3^{2-} by washing.

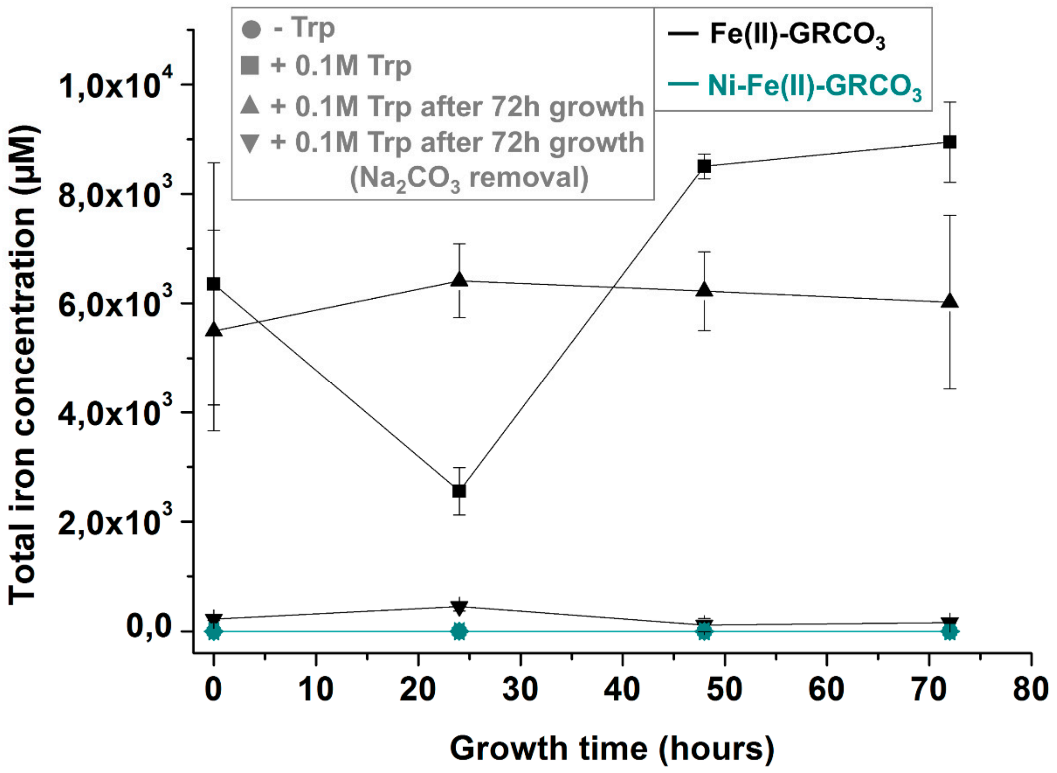

<u>Supplementary information Figure S5</u>: Monitoring of total dissolved iron of both Fe(II)-GRCO$_3$ (black) and Ni(II)-Fe(II)-GRCO$_3$ (green) samples at 0h, 24h, 48h and 72h after 0.1M addition either 5 minutes after co-precipitation (filled squares), 72h hours after co-precipitation (filled up-triangles) or 72h hours after co-precipitation upon Na$_2$CO$_3$ removal (filled down-triangles) and without tryptophan addition (control, filled circles).

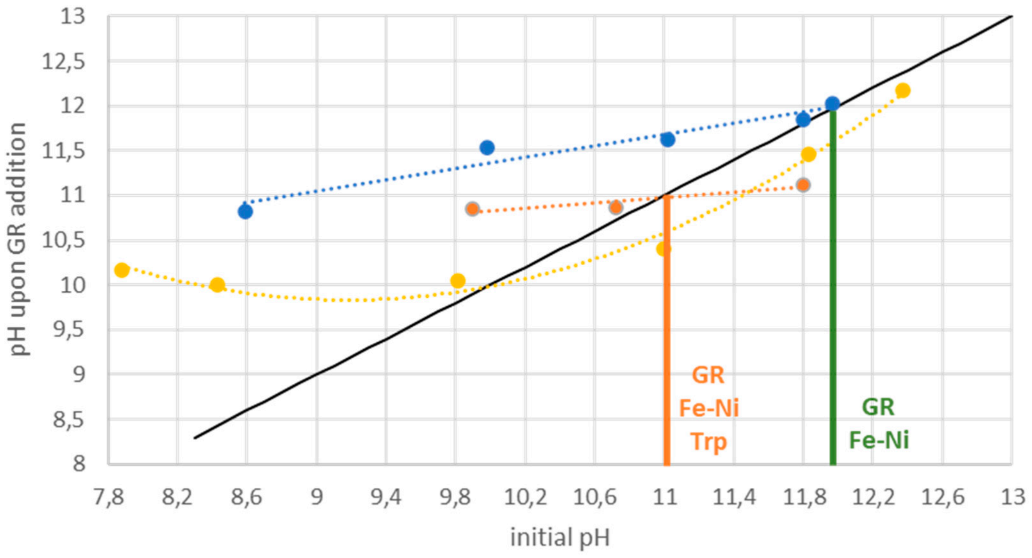

<u>Supplementary information Figure S6</u>: Point of Zero Charge (PZC) determination of various GRCO$_3$ samples via resuspensions of dry pellets of GRCO$_3$ using the method described in *Angela et al.* The PZC of Ni(II)-Fe(II)-GRCO$_3$ decreases from approximately 12 down to approximately 11 after functionalization with tryptophan, suggesting that tryptophan neutralized part of the positive charges at the surface of the crystals, supposedly during an electrostatic interaction.

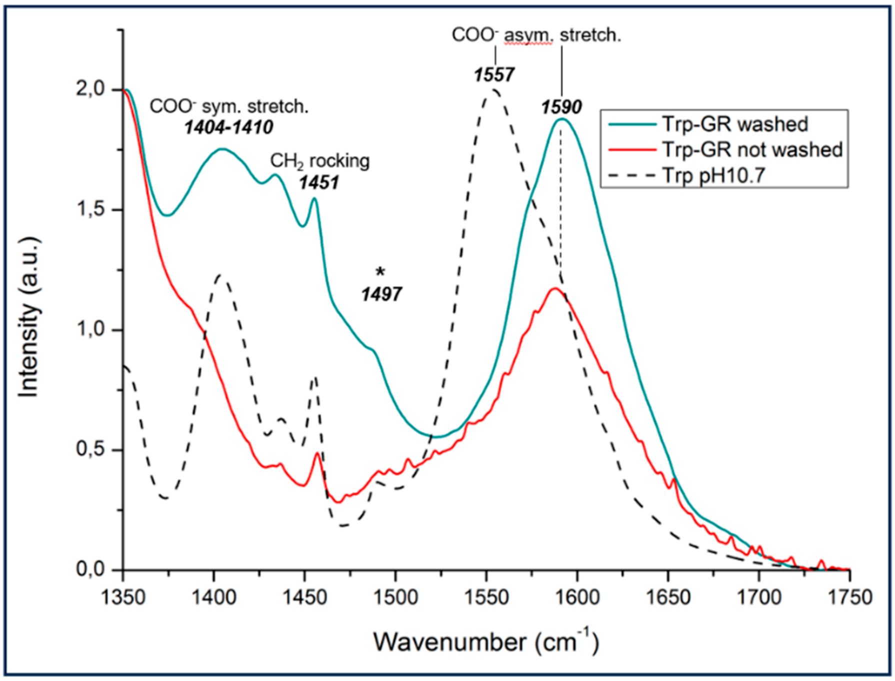

<u>Supplementary information Figure S7</u>: FT-Infrared spectra of tryptophan in the 1350-1750 cm^{-1} region during interaction with Ni(II)-Fe(II)-$GRCO_3$ with (green spectrum) or without (red spectrum) washing of the excess tryptophan in solution prior to drying, and compared to the control spectrum of anionic tryptophan (black dashed spectrum).

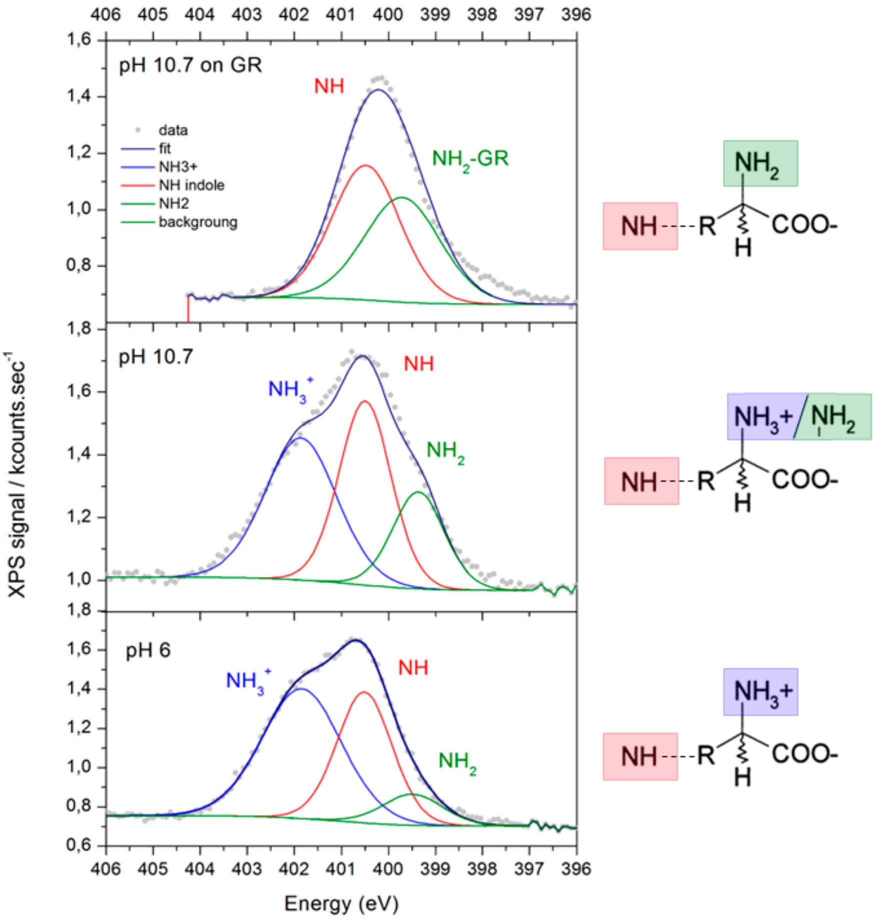

<u>Supplementary information Figure S8</u>: X-ray Photoelectron Spectroscopy (XPS) speciation of tryptophan-associated nitrogen in control zwitterionic (bottom spectrum) and anionic (middle spectrum) tryptophan compared to trytophan interacting with Ni(II)-Fe(II)-$GRCO_3$ (top spectrum), displaying the protonated state of tryptophan's amine function in these conditions.

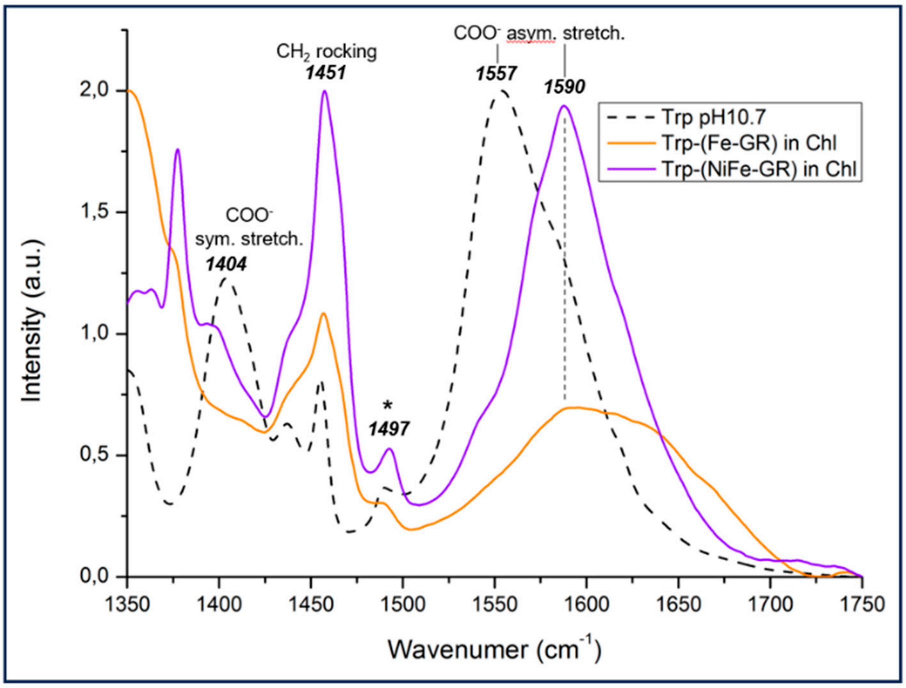

<u>**Supplementary information Figure S9:**</u> **FTIR spectra of tryptophan (black) in the 1350-1750 cm^{-1} region** during interaction with Ni(II)-Fe(II)-$GRCO_3$ (purple) or Fe(II)-$GRCO_3$ (orange) upon retrieval of corresponding functionalized suspensions in chloroform during hydrophobicity tests.
